# Supplementary material for: A time-resolved multi-omic atlas of the developing mouse stomach
Source: Nat Commun. 2018 Nov 21;9:4910. doi: 10.1038/s41467-018-07463-9 (PMC6249217; doi:10.1038/s41467-018-07463-9)
Supplement: Supplementary file 1 — Supplementary Information [file 41467_2018_7463_MOESM1_ESM.pdf]

A Time-resolved Multi-omic Atlas of the Developing Mouse Stomach

Li et al.

Supplementary Figure 1

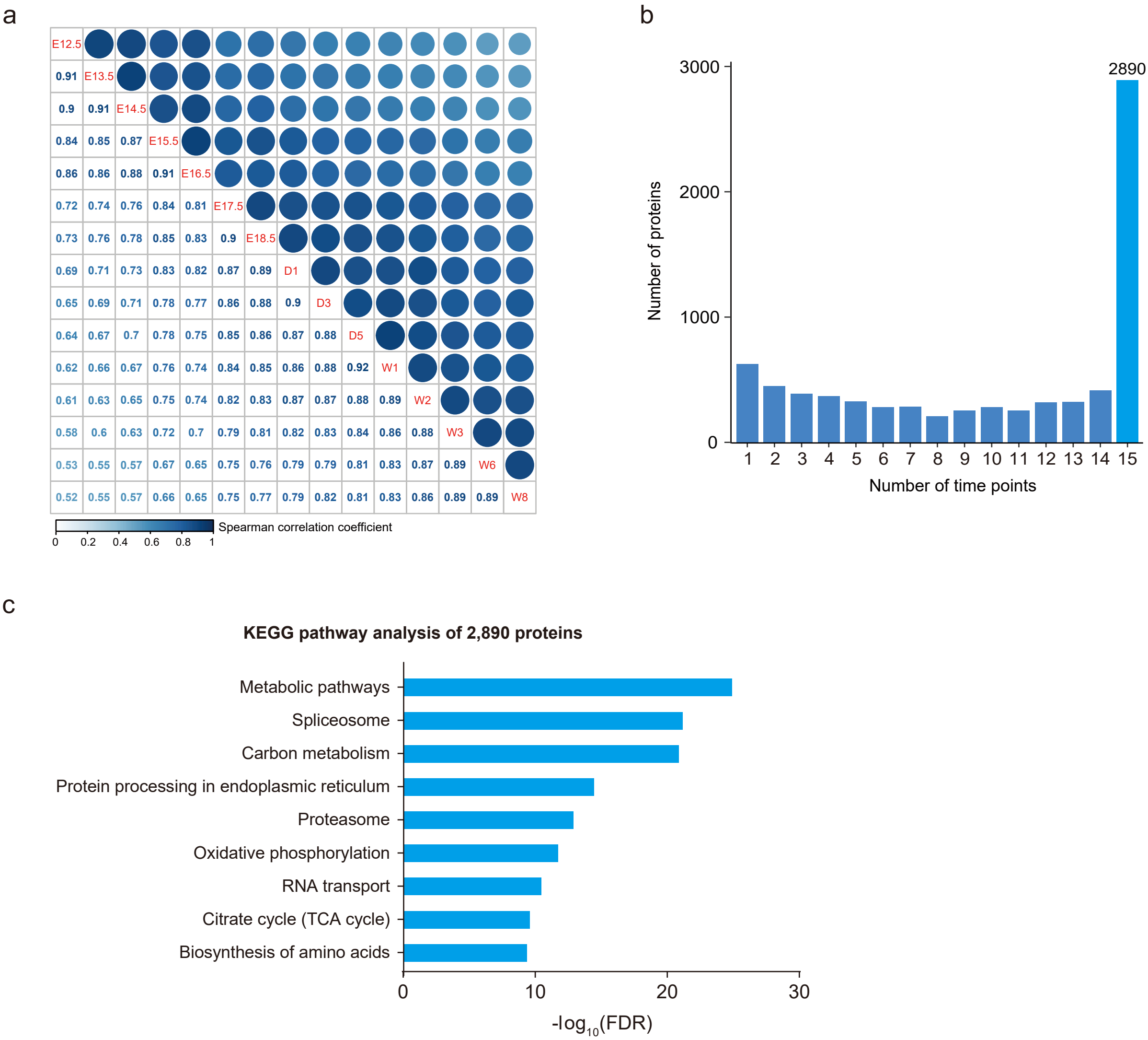

Supplementary Figure 1. Analysis based on 15 timepoints

(a) Correlation analysis of experiments between 15 timepoints. (b) Number of proteins identified in 1-15 timepoints. DodgerBlue: proteins identified in all 15 timepoints. (c) KEGG pathway analysis of 2,890 core proteins identified in all 15 timepoints.

Supplementary Figure 2

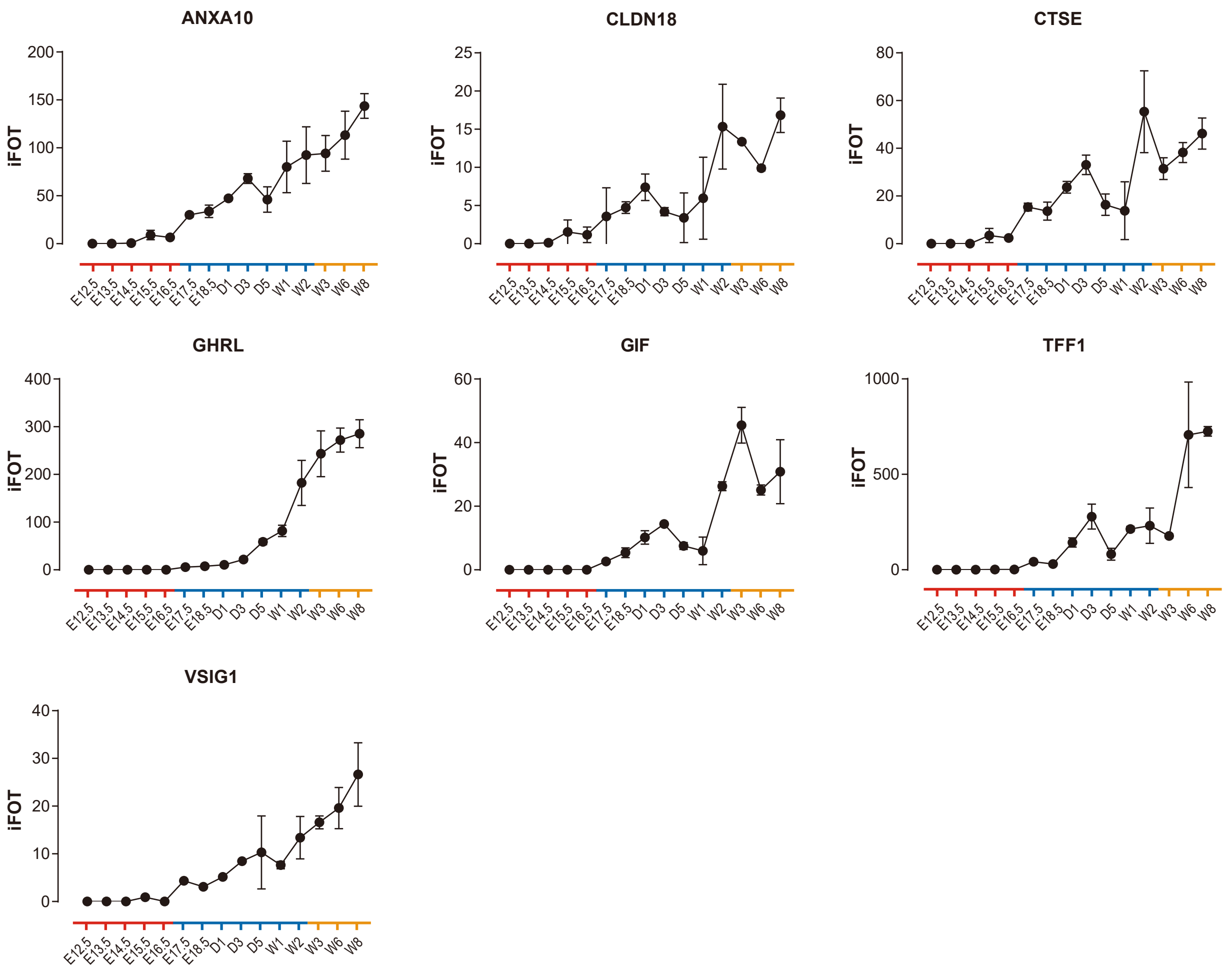

Supplementary Figure 2. Proteomic profiles of seven stomach proteins

Protein dynamics of seven gastric proteins: ANXA10, CLDN18, CTSE, GHRL, GIF, TFF1 and VSIG1. Error bars are created by mean ± SD (standard deviations) of three replicates. Horizontal color bars: red, Ph1; blue, Ph2; and orange, Ph3.

Supplementary Figure 3

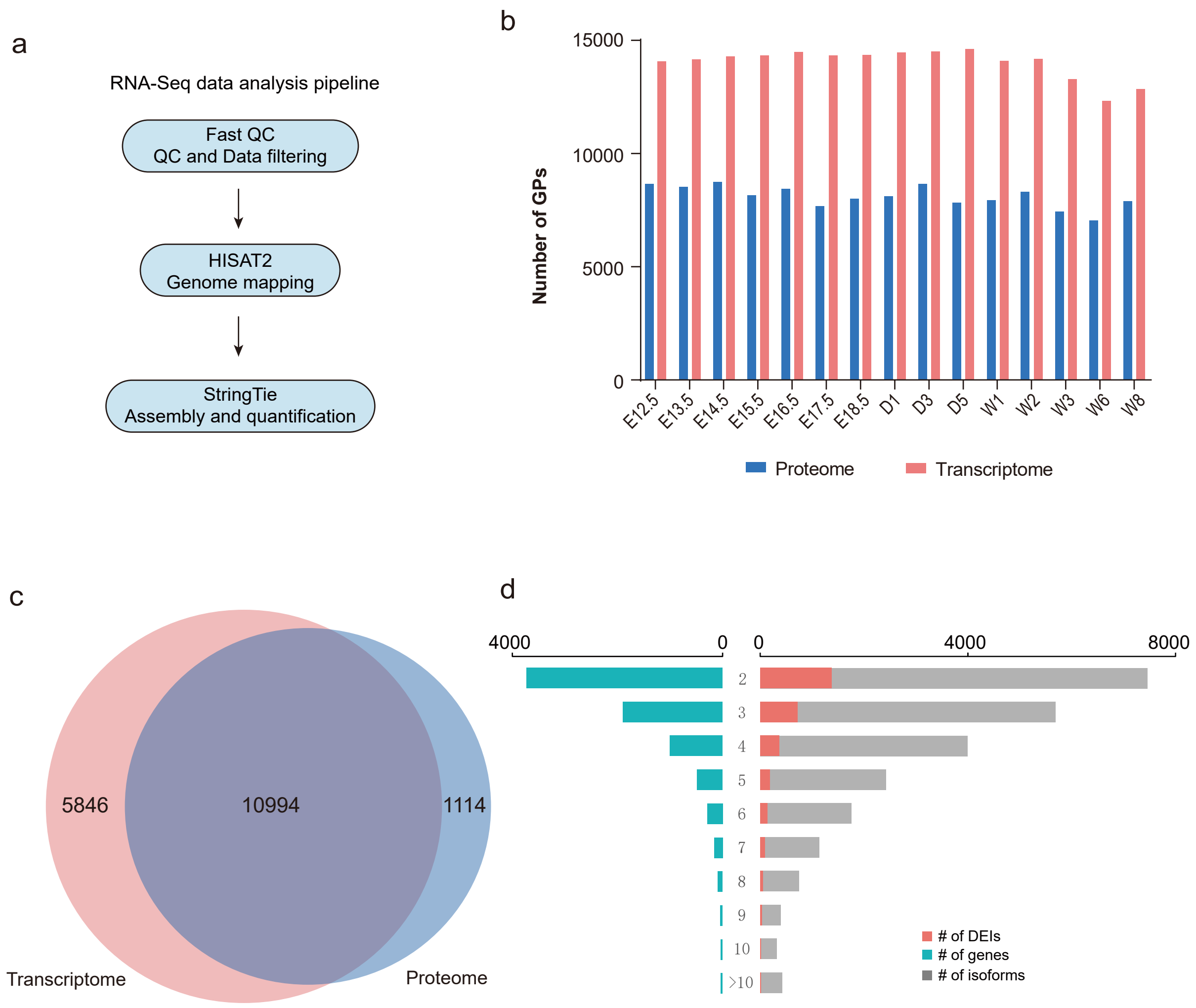

Supplementary Figure 3. Comparison of proteins and mRNA

(a) RNA-Seq data analysis pipeline. (b) The number of genes identified in 15 transcriptomes compared to proteomes.
(c) Venn diagram showing the number of genes and proteins identified in proteome and transcriptome.
(d) Distributions of genes, their isoforms, and differentially regulated isoforms (ANOVA, FDR < 0.01).
Abbreviations: DEIs, Differentially Expressed Isoforms.

Supplementary Figure 4

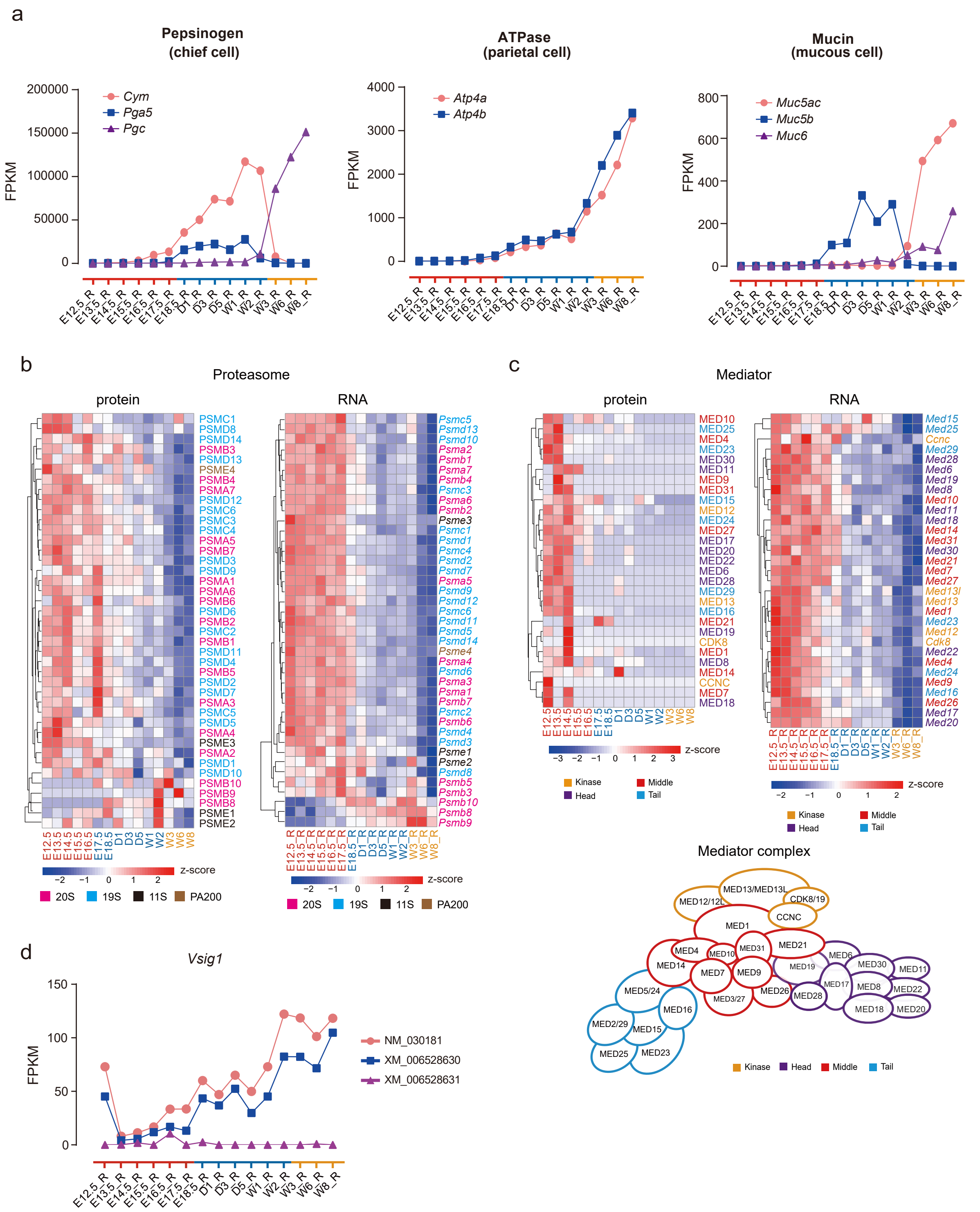

Supplementary Figure 4. Transcriptomic profiles of three protein families and two protein complexes

(a) Transcriptomic changes of eight gastric markers in three protein families. (b) The dynamic profiles of the proteasome complex and its members at the RNA and protein levels. The changes in the components in the four subunits are illustrated in four colors. (c) The changes in the mediator complex and its members at the RNA and protein levels. The four subunits of the mediator are illustrated in four colors. (d) The expression patterns of the Vsig1 transcripts. Horizontal color bars: red, Ph1; blue, Ph2; and orange, Ph3.

Supplementary Figure 5

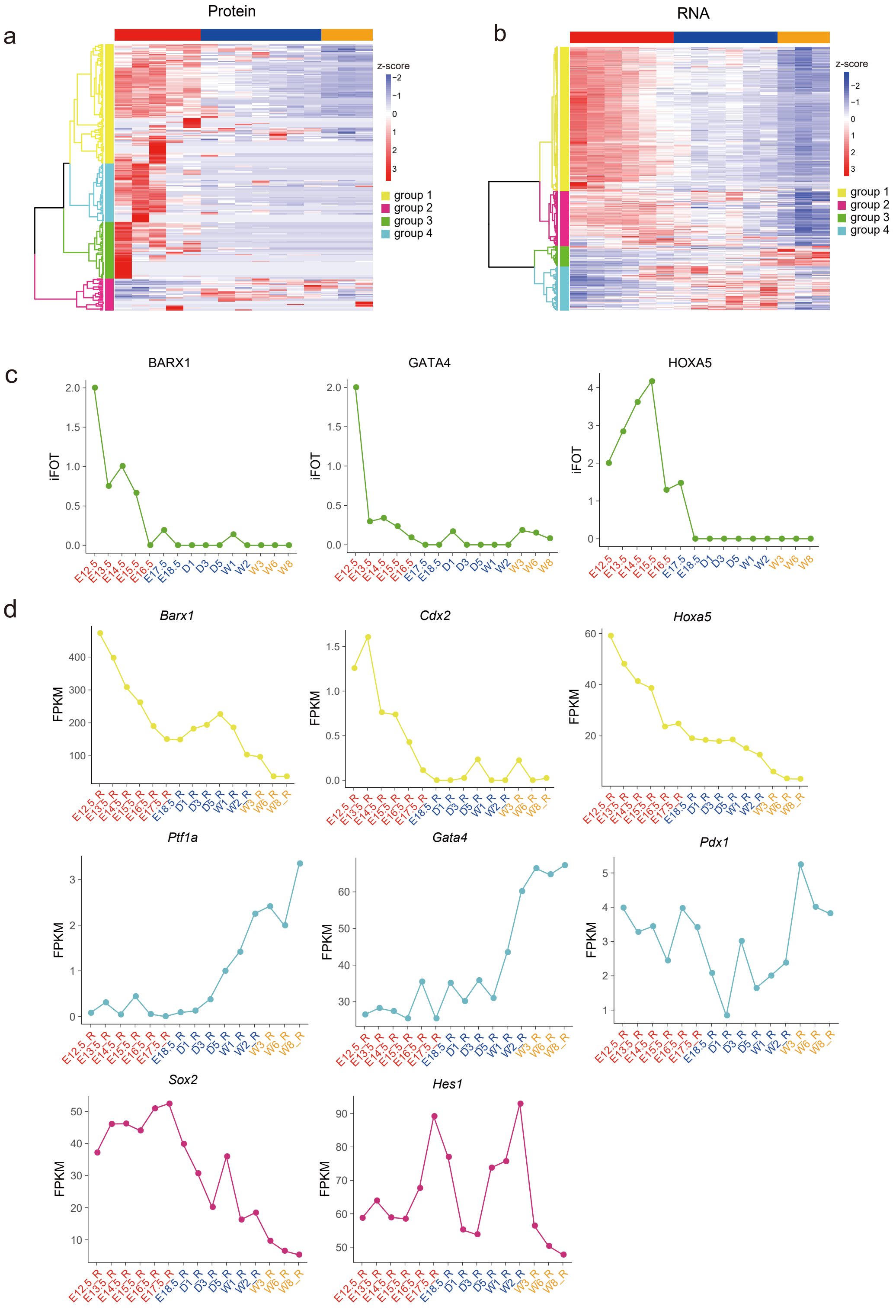

Supplementary Figure 5. Proteomic and transcriptomic analyses of transcription factors

(a) Unsupervised Ward's hierarchical clustering of 241 transcription factors (TFs) at the level of protein abundances. (b) Unsupervised Ward's hierarchical clustering of 724 TFs at the level of mRNA abundances. (c) Proteomic profiles of three TFs: BARX1, GATA4 and HOXA5. (d) Transcriptomic profiles of eight TFs: Barx1, Cdx2, Hoxa5, Ptf1a, Gata4, Pdx1, Sox2 and Hes1. Yellow, group 1; Deeppink, group 2;Green, group 3; Cyan, group 4.
