# Supplementary material for: A time-resolved multi-omic atlas of the developing mouse stomach
Source: Nat Commun. 2018 Nov 21;9:4910. doi: 10.1038/s41467-018-07463-9 (PMC6249217; doi:10.1038/s41467-018-07463-9)
Supplement: Supplementary file 3 — Description of Additional Supplementary Files [file 41467_2018_7463_MOESM3_ESM.pdf]

Description of Additional Supplementary Files

File Name: Supplementary Data 1
Description: Proteins identified in 15 timepoints

File Name: Supplementary Data 2
Description: One-way ANOVA analyses of quantified proteins

File Name: Supplementary Data 3
Description: Transcriptome data analysis

File Name: Supplementary Data 4
Description: Transcriptomic analysis of isoforms during stomach development

File Name: Supplementary Data 5
Description: A list of 60 novel splice-junction peptides and their relative abundances at 15 timepoints

File Name: Supplementary Data 6
Description: Tandem mass spectra of 60 novel splice-junction peptides

File Name: Supplementary Data 7
Description: Analyses of transcription factors at the protein and mRNA levels

File Name: Supplementary Data 8
Description: Comparisons of stomach development and DGC
