# Supplementary material for: A time-resolved multi-omic atlas of the developing mouse stomach
Source: Nat Commun. 2018 Nov 21;9:4910. doi: 10.1038/s41467-018-07463-9 (PMC6249217; doi:10.1038/s41467-018-07463-9)

Supplementary Data 6. Tandem mass spectra of 60 novel splice-junction peptides

1. AVIWIVIR

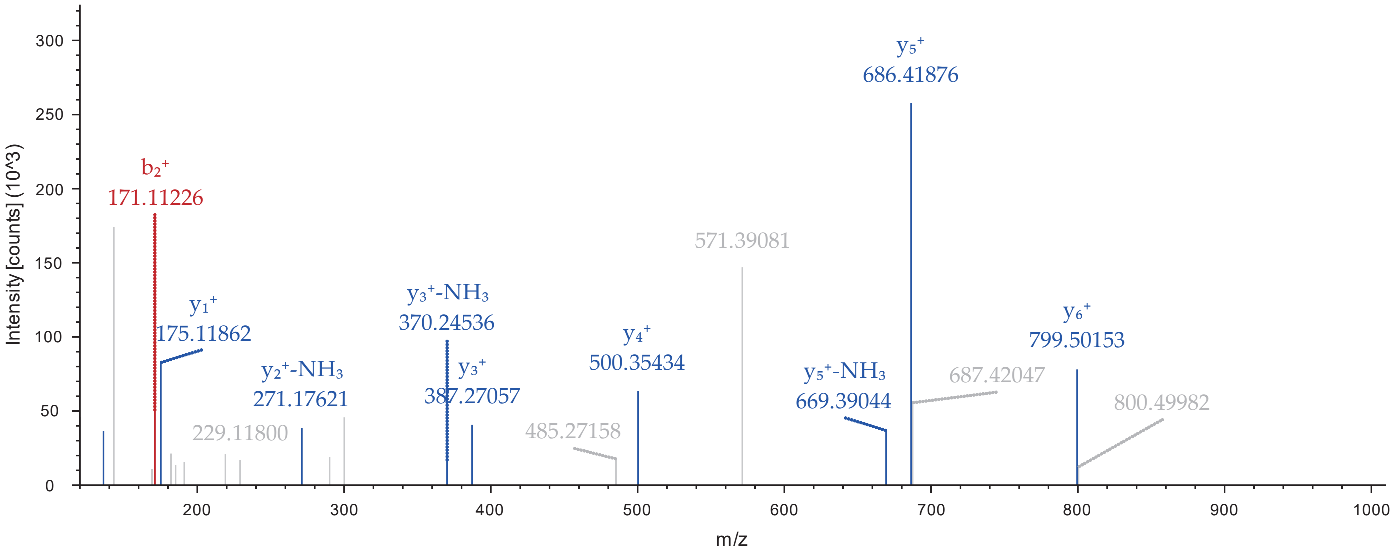

2. LEDLVQESMEEK

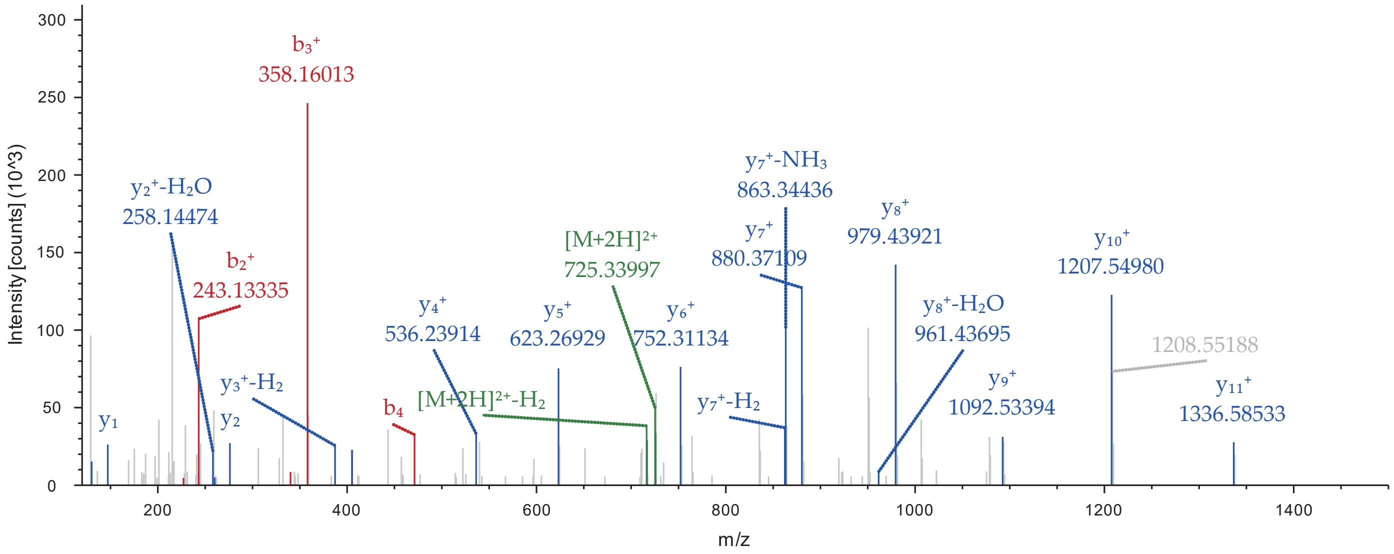

3. IPVSNLDEESR

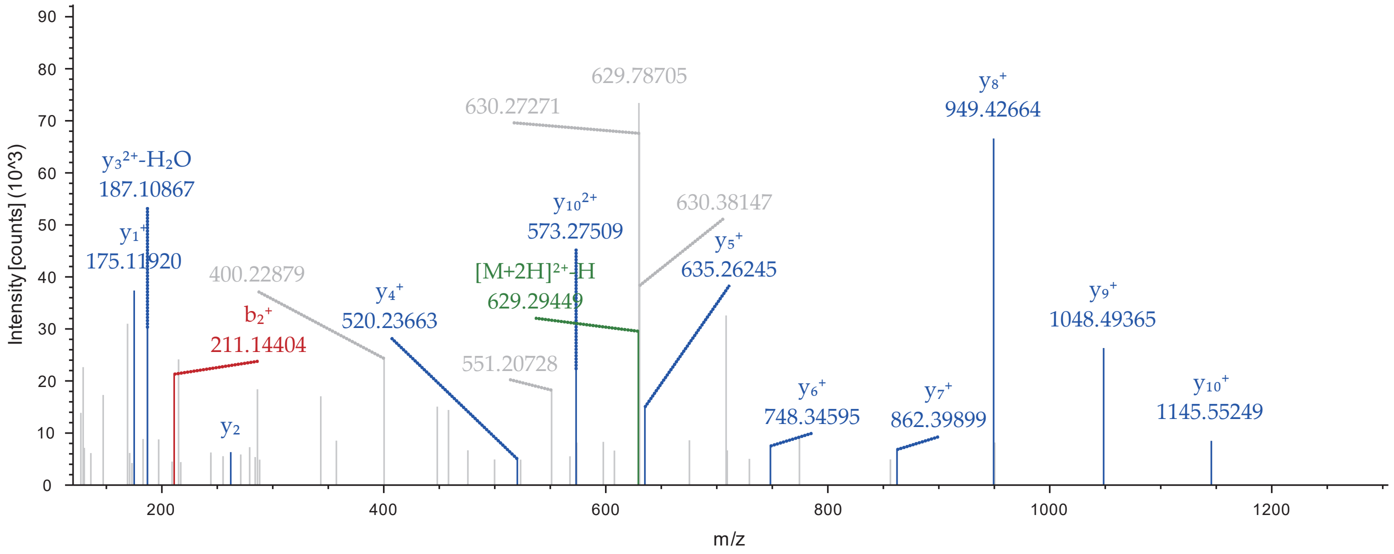

4. NEPCHIIQILEIMSAVR

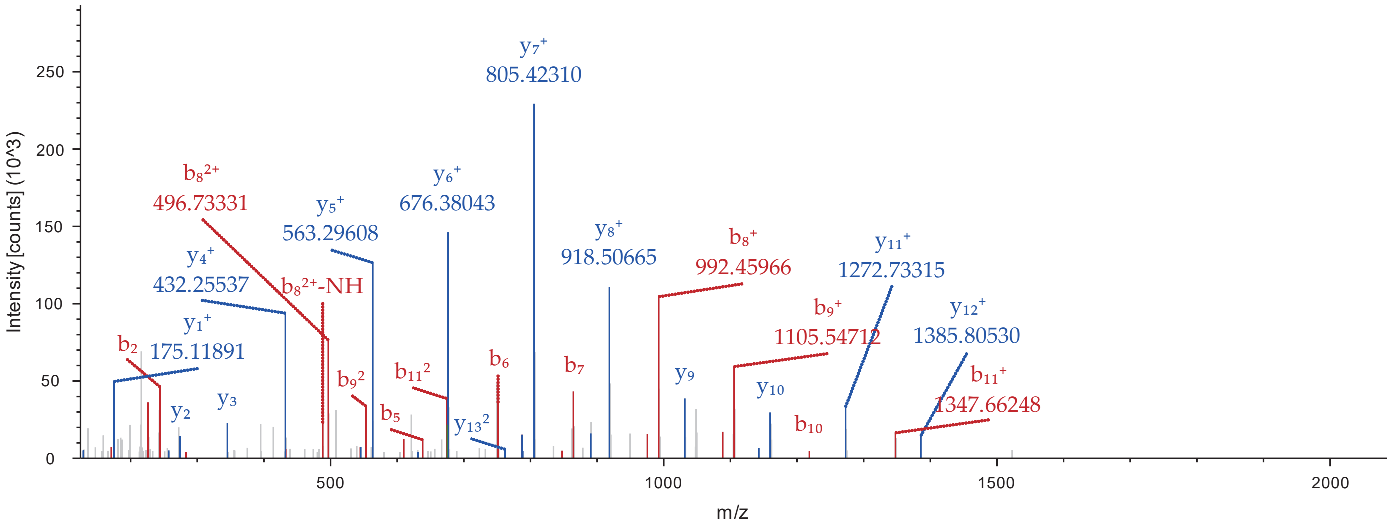

5. LSGPLISDFFAK

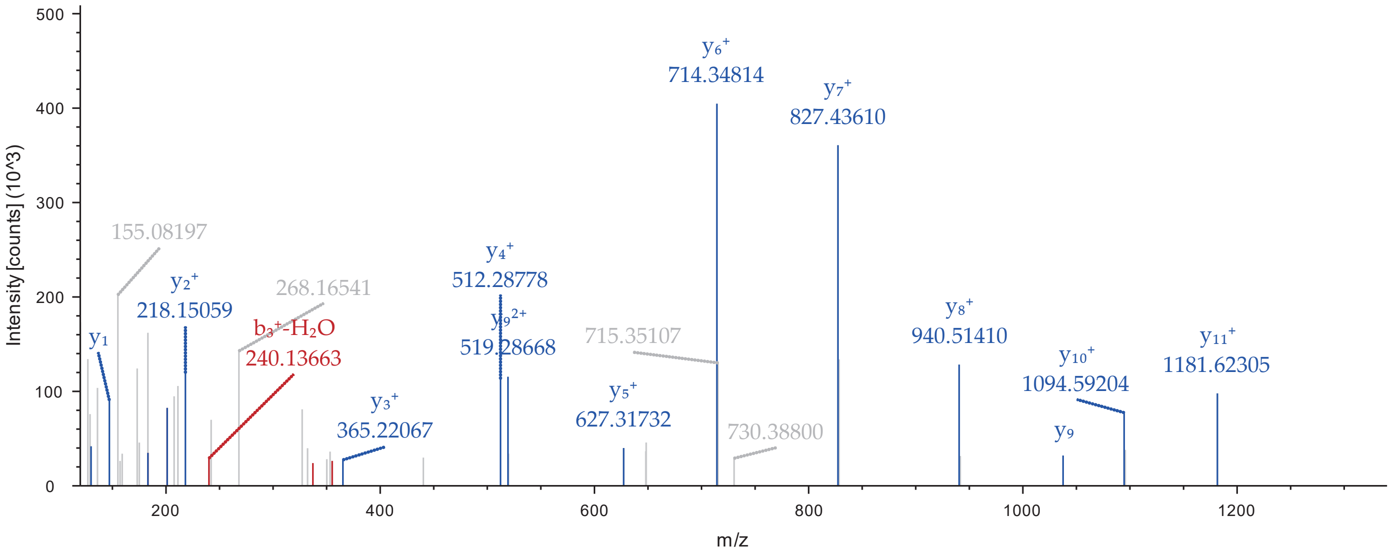

6. NIPIVVLPLTDNWVVK

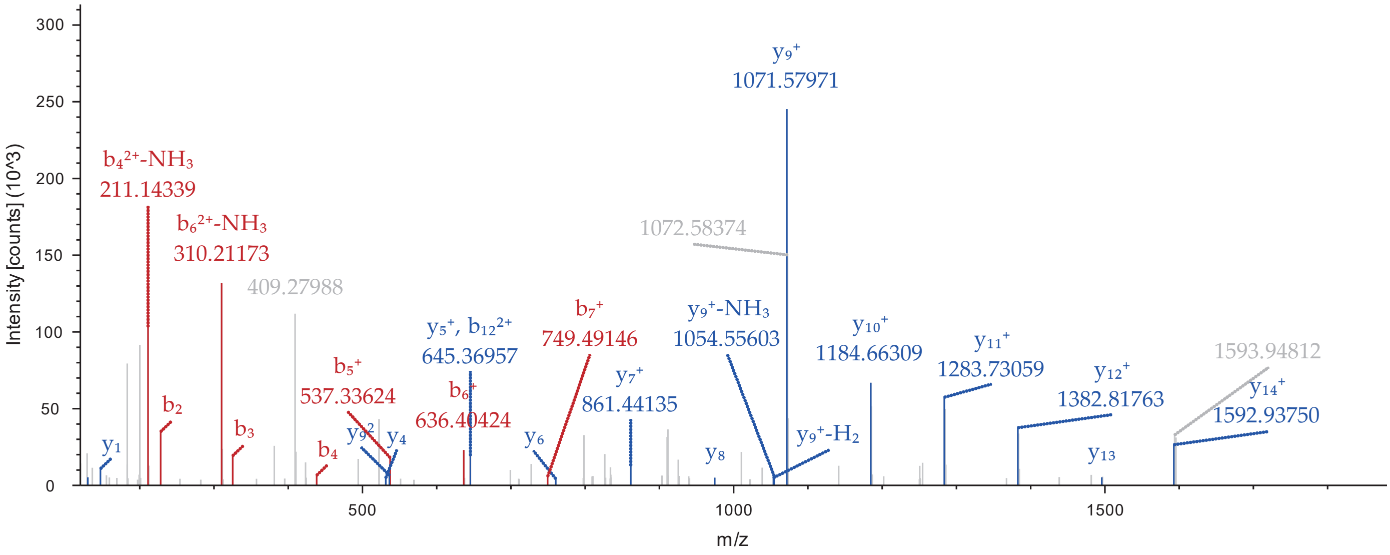

7. LLGSLTSVFVR

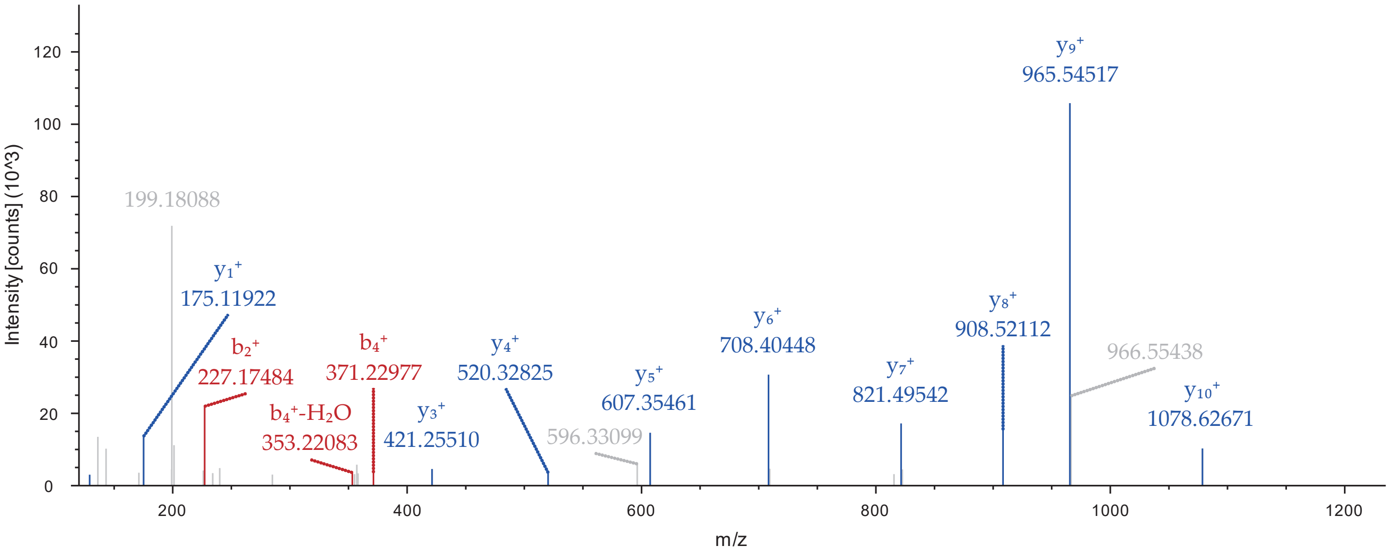

8. ETSLLGLPVAAVPGALSPLAIPNAAAAAAAAAAGR

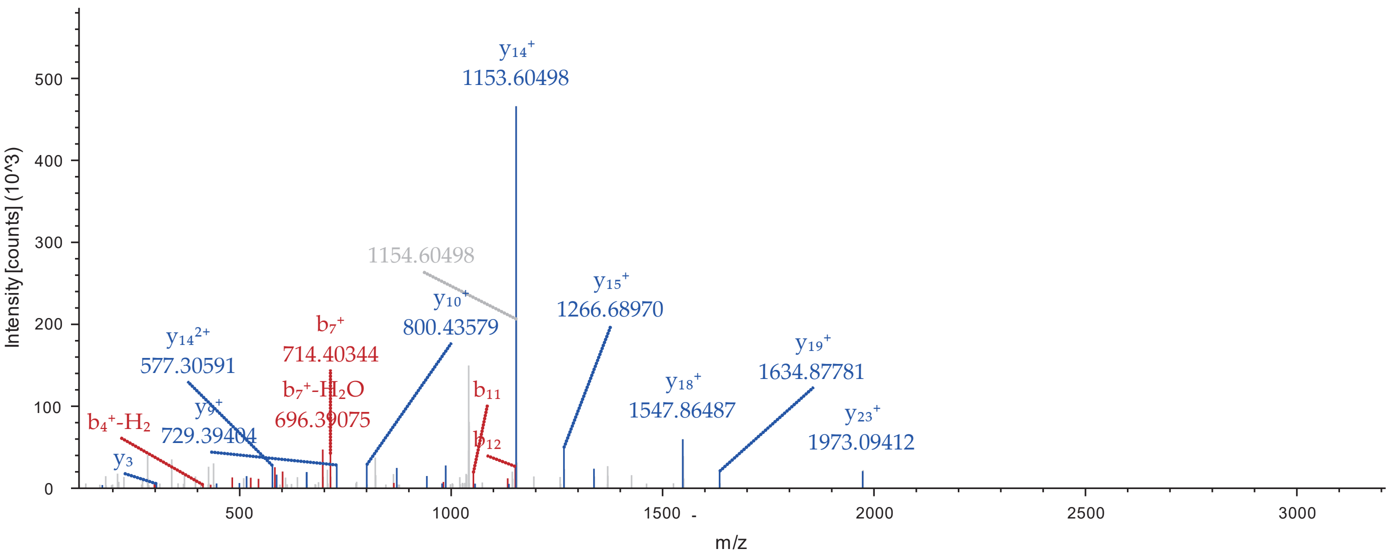

9. YPPGSMATLQLLR

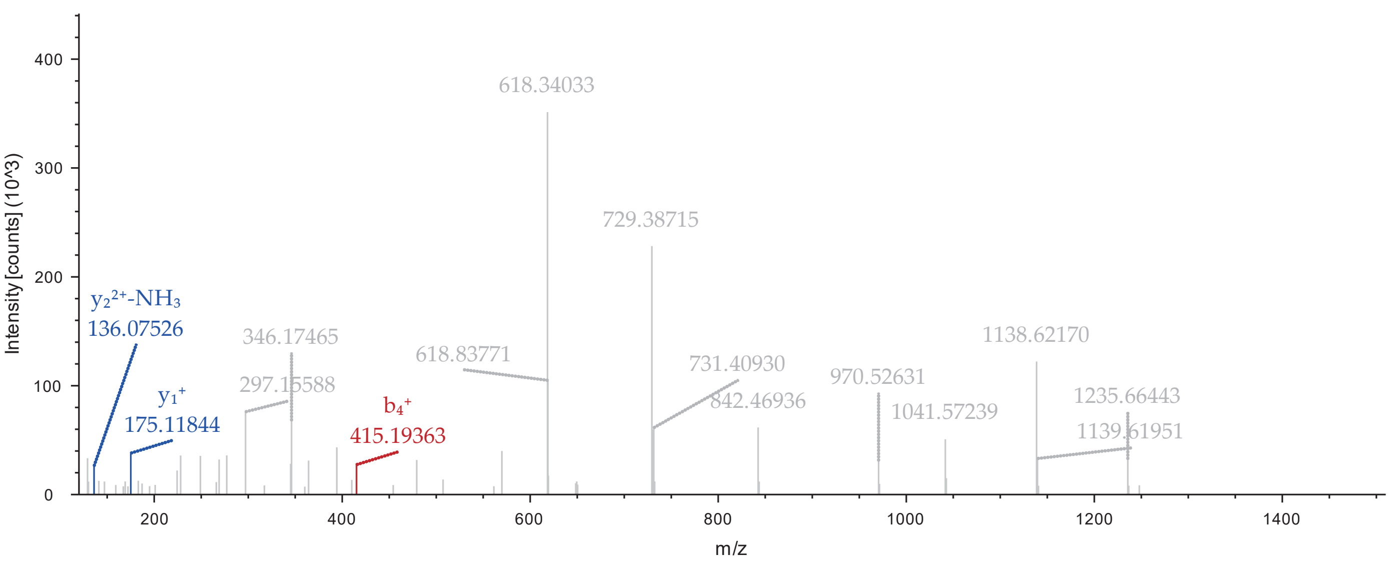

10. VEMAETDQR

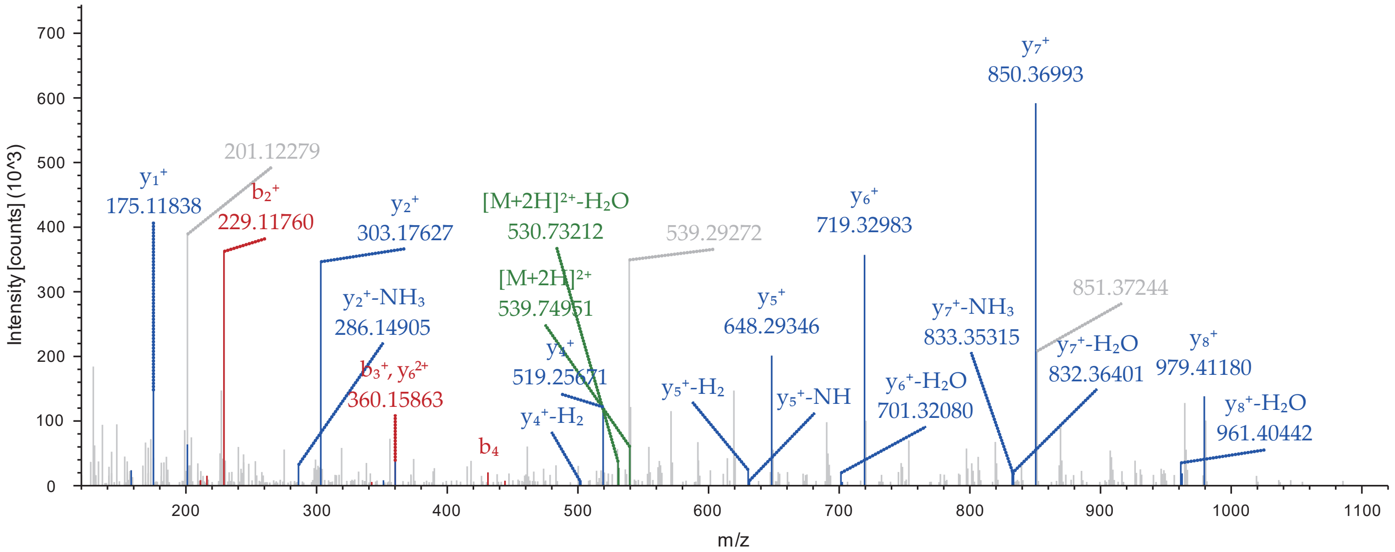

11. ESHLQEADSQIGR

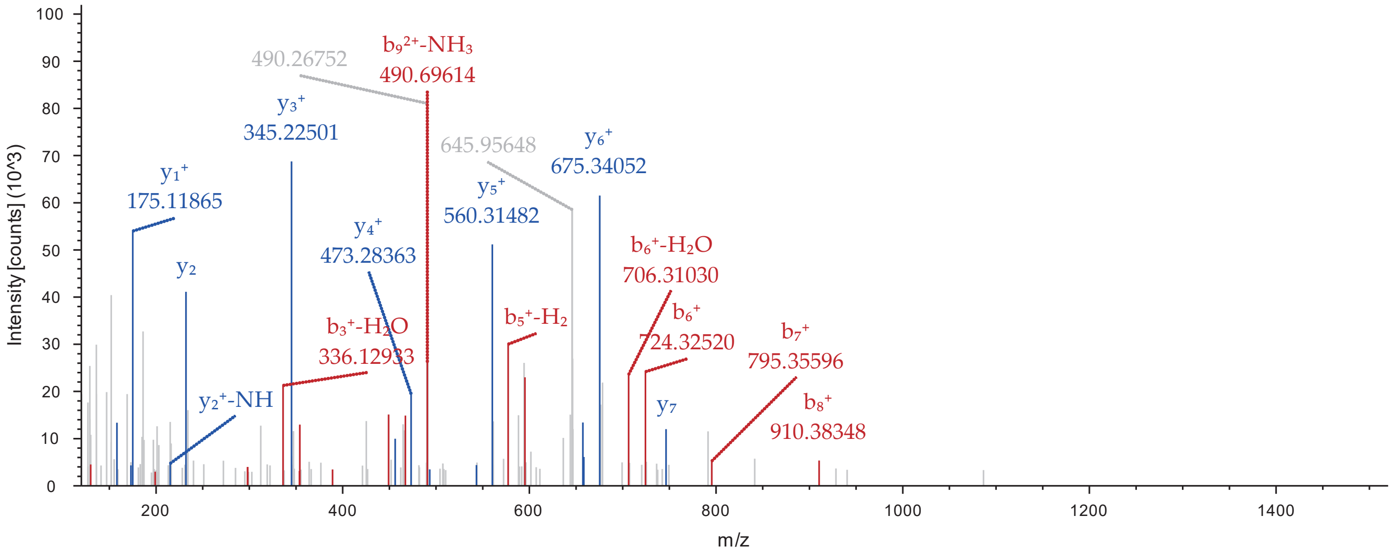

12. GAVGLSDVVIPENPEAYVK

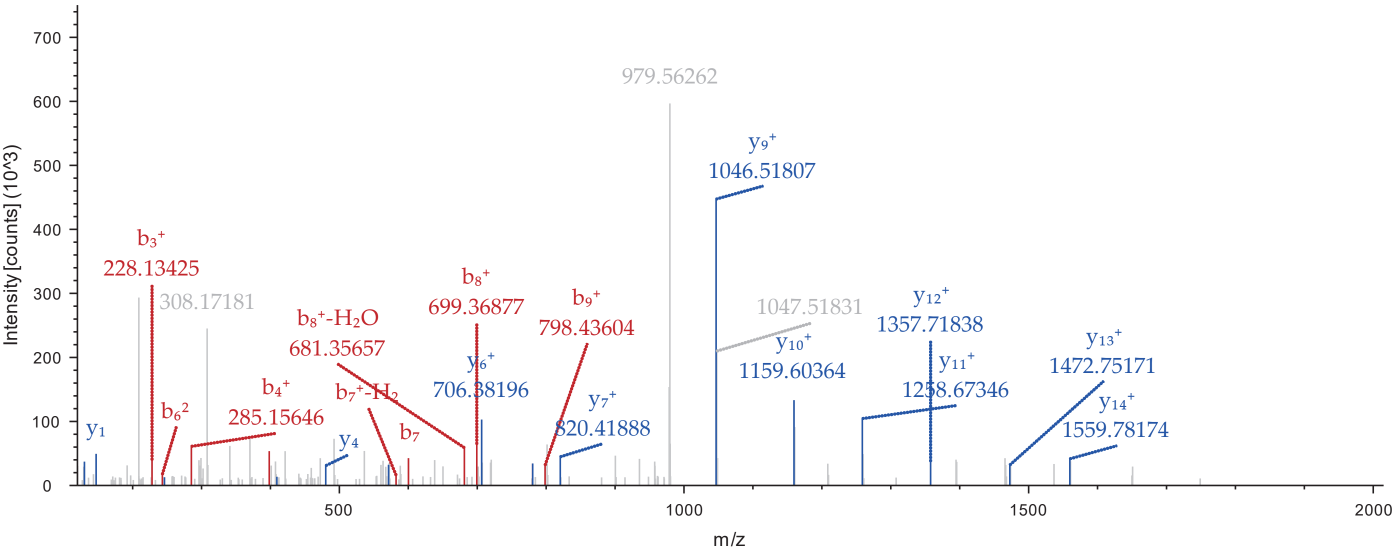

13. HSSPLAHQAQQGSPGPSGALESMVK

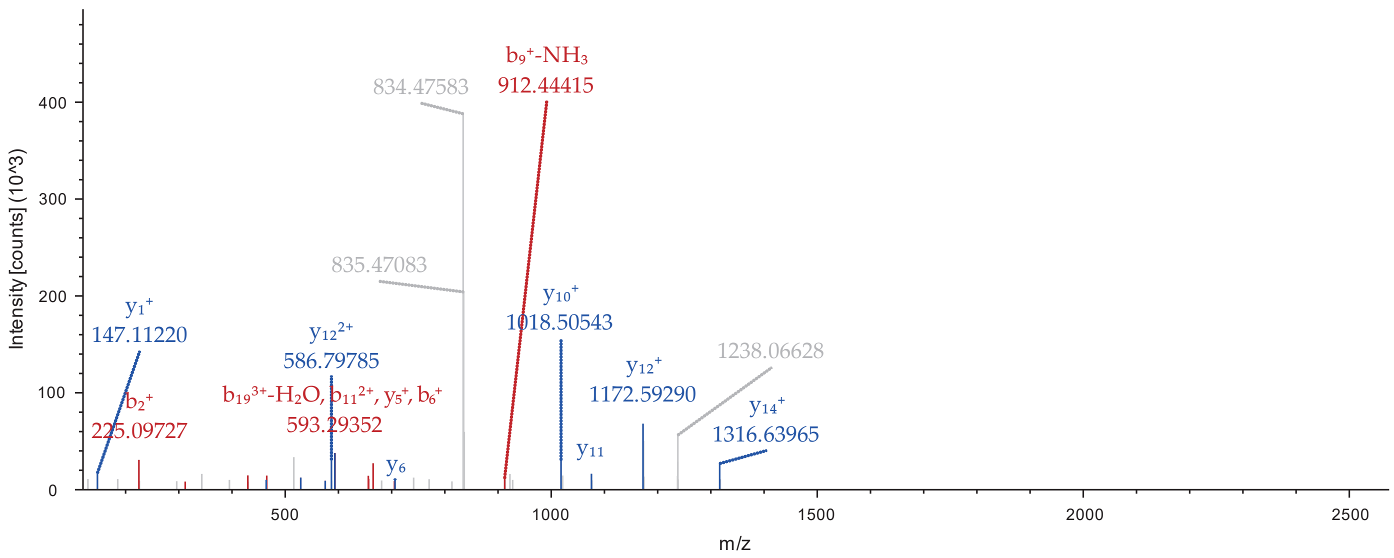

14. SASLSGVQAR

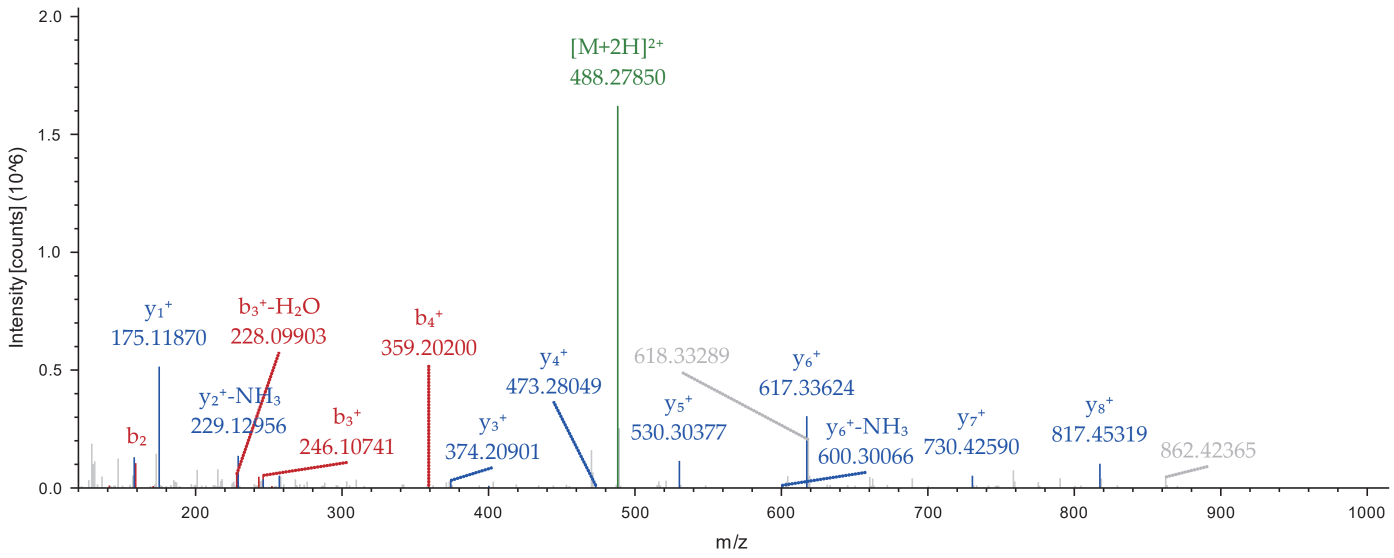

15. SFVSAEFFEMLEK

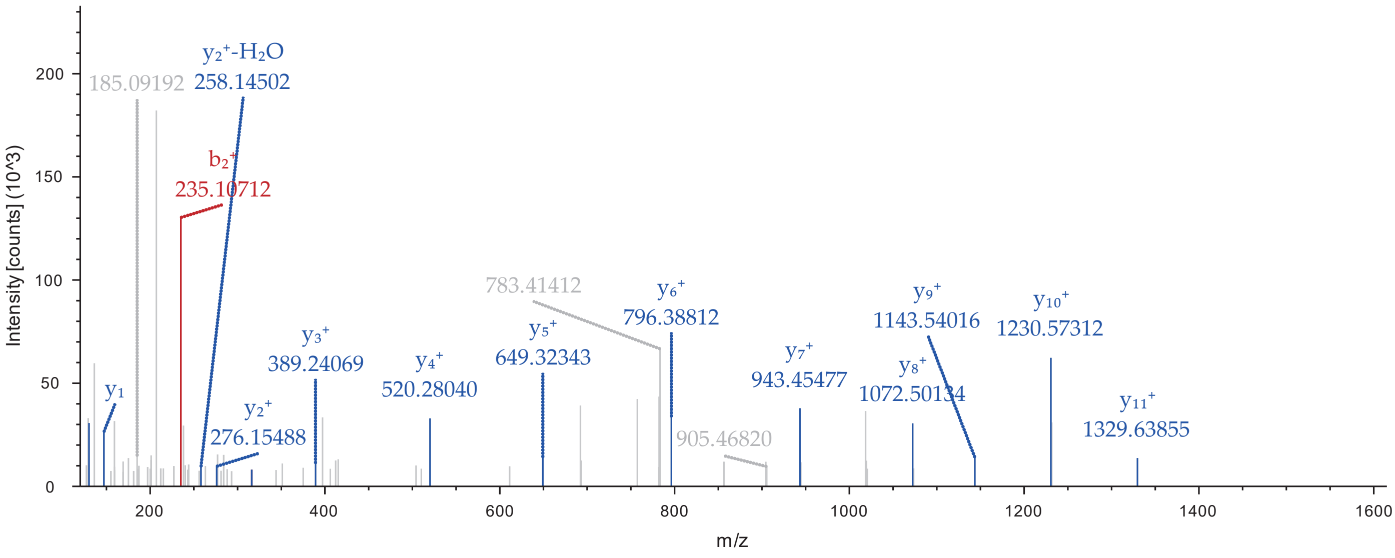

16. CSWTSQIYYSEGGQASAIGQFK

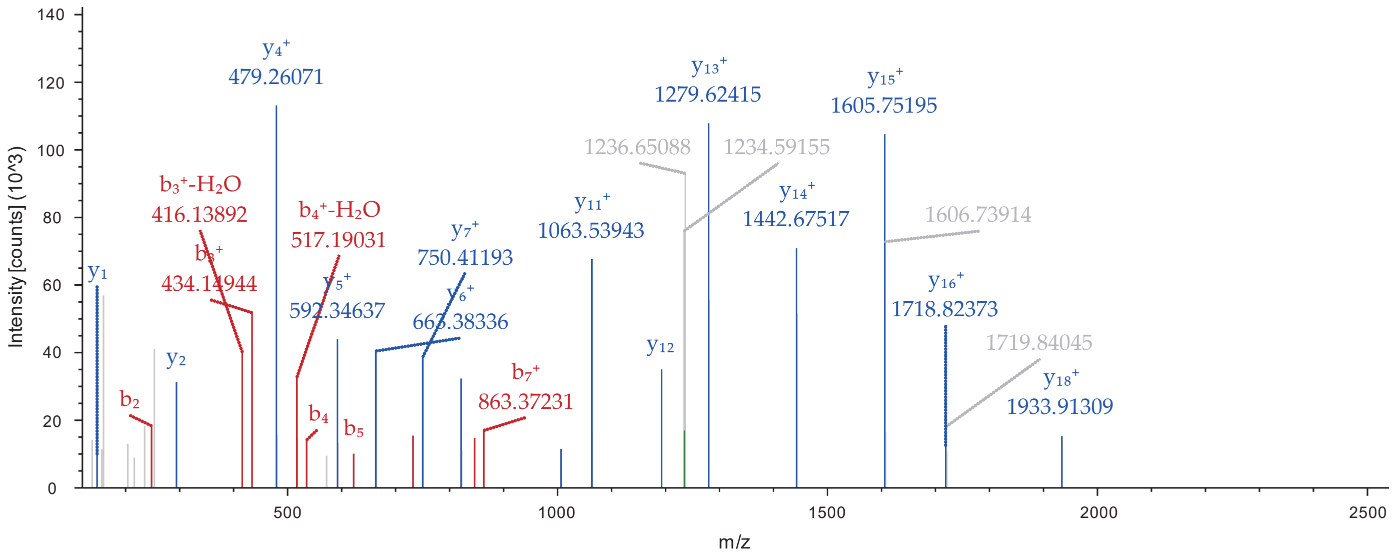

17. EMEEPISHVSHPK

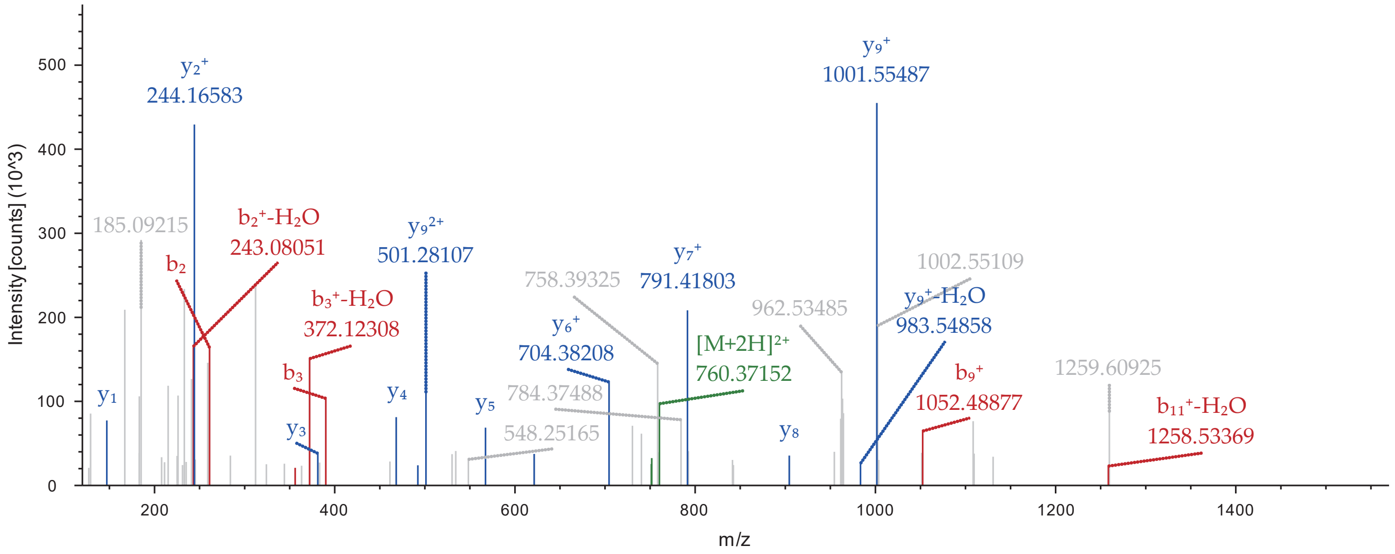

18. SATVSPQPSHHSLSAGPDLTESEK

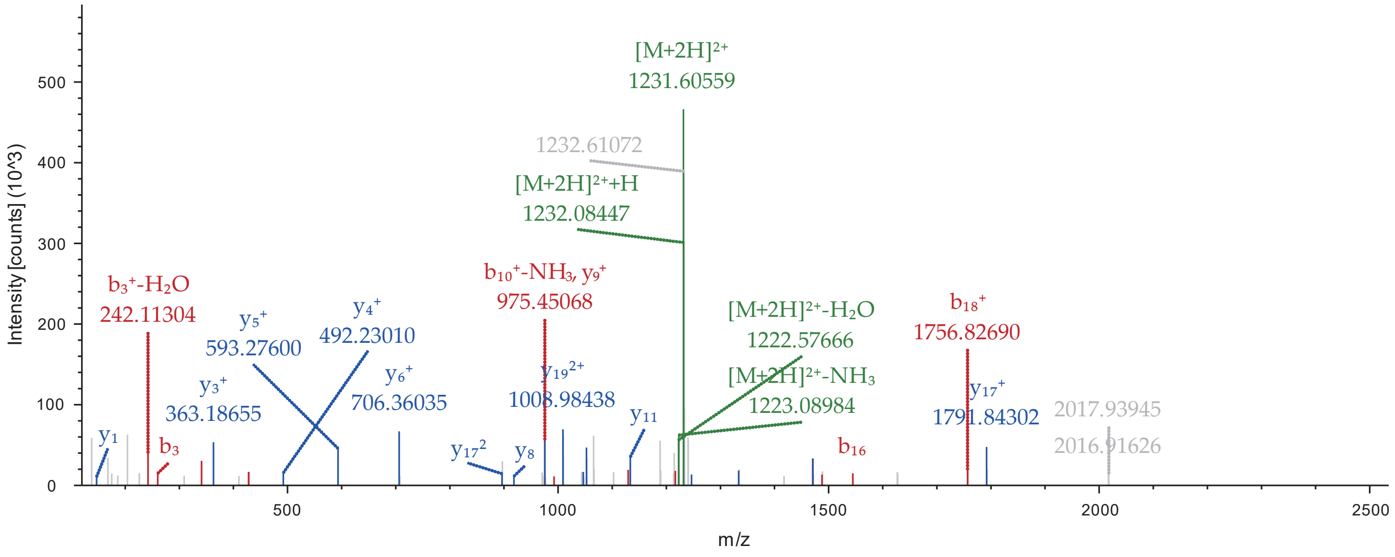

19. GWEMDSTPEGAAYFINHNER

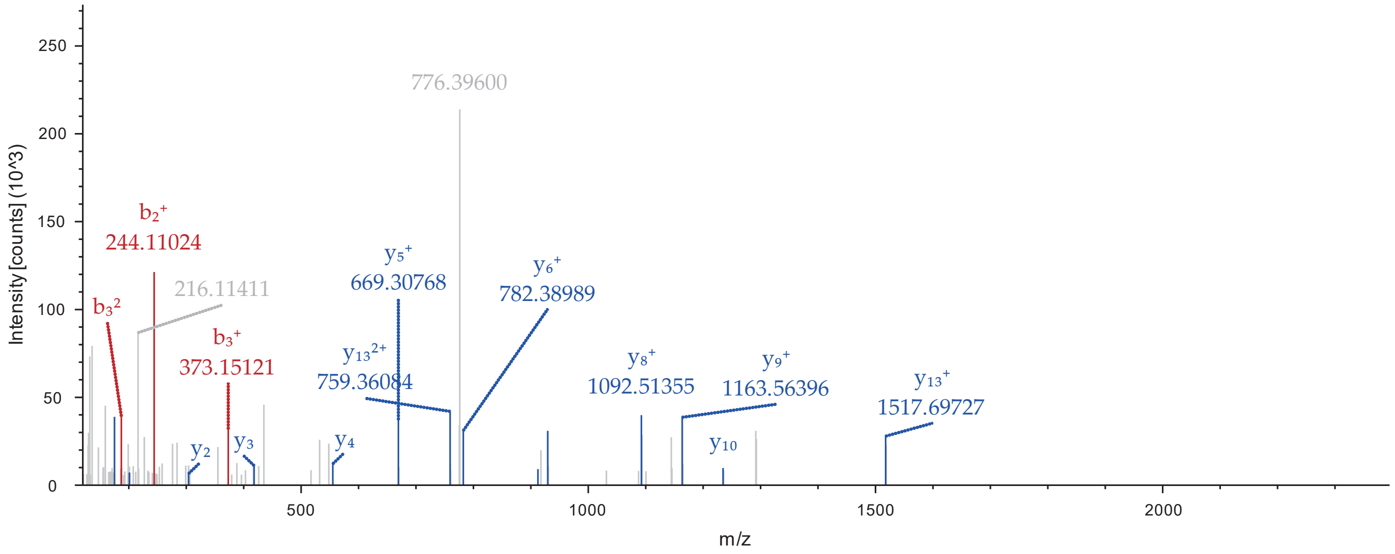

20. VGPVSTVGVTDPK

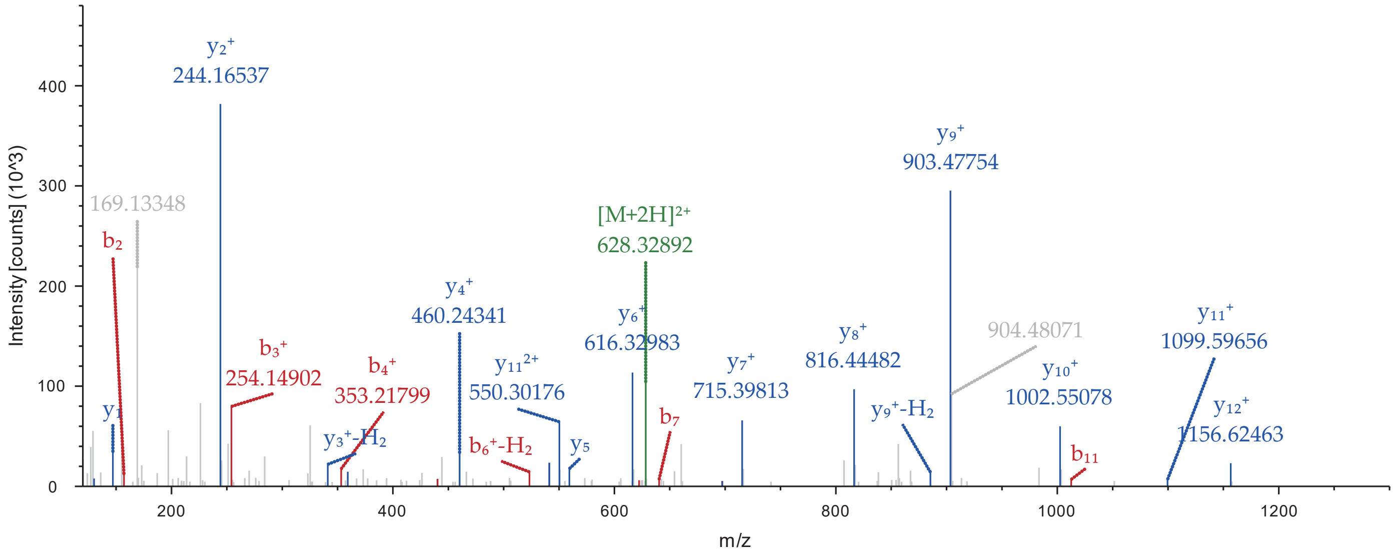

21. QNLLQAAGNVGQASGELLQQIGESDTDPHFQMCVPR

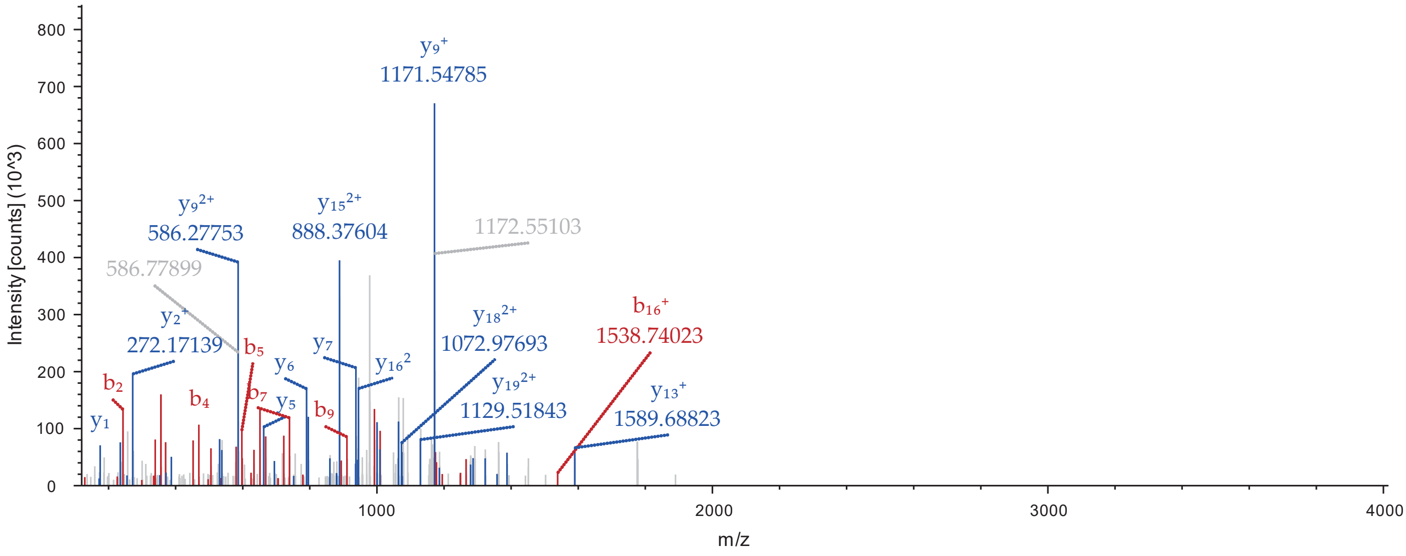

22. MTEEEVEMLVAGHEDSNGCINYEAFVR

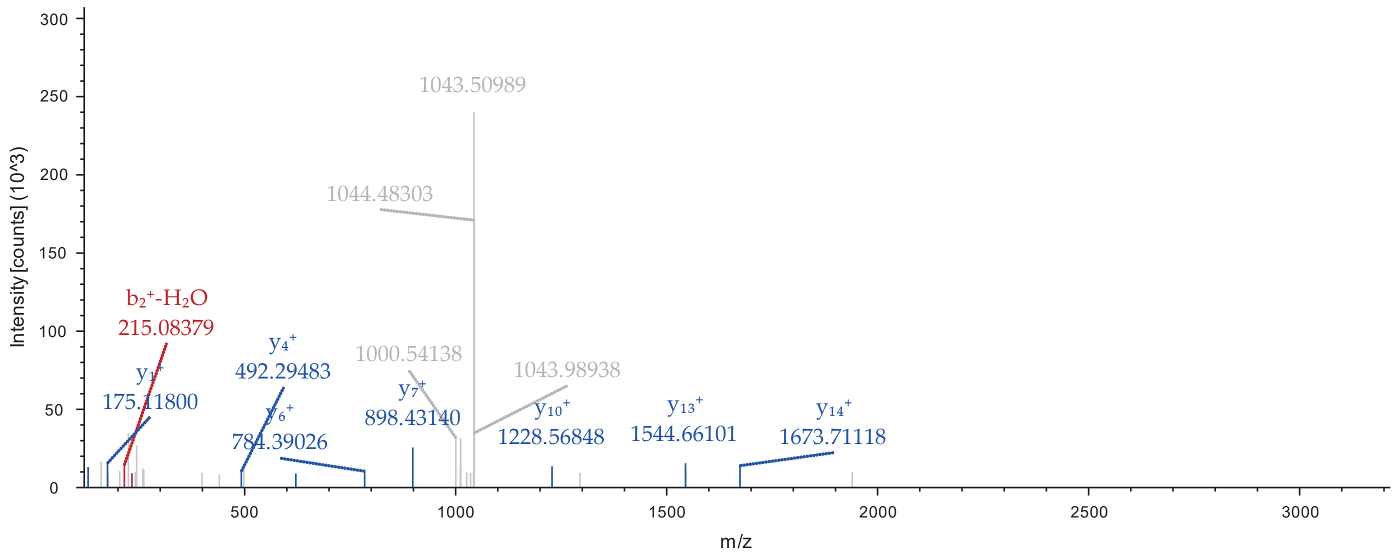

23. IGLVATVK

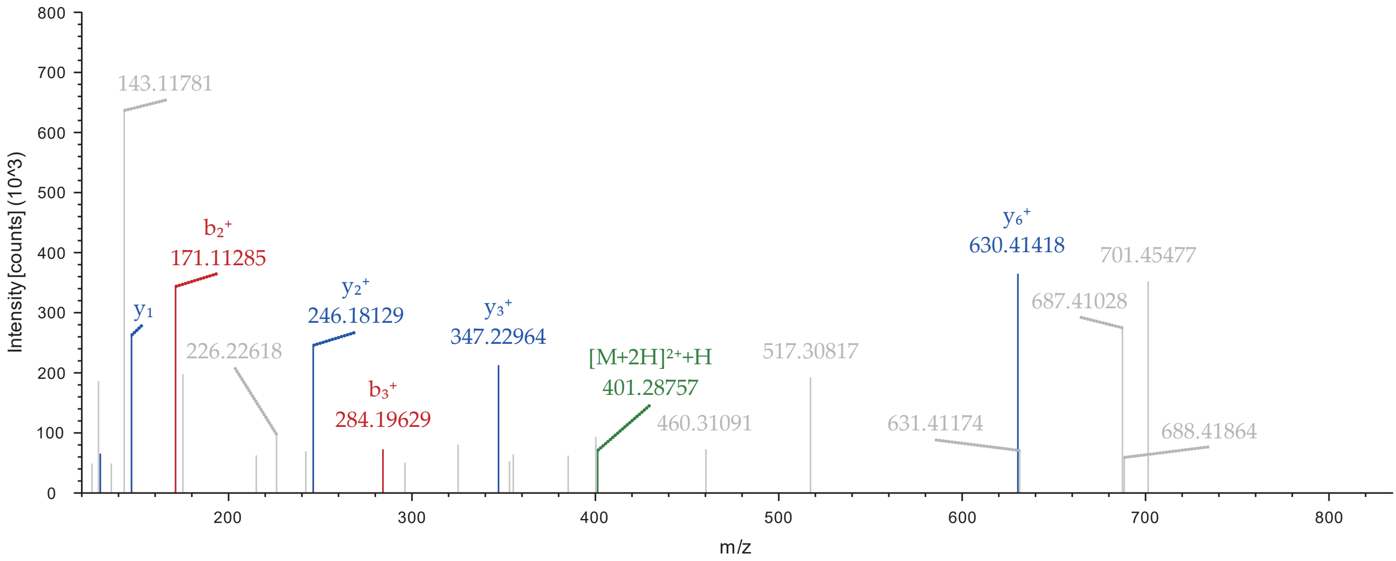

24. EDAGVVCSGTDSGLAVR

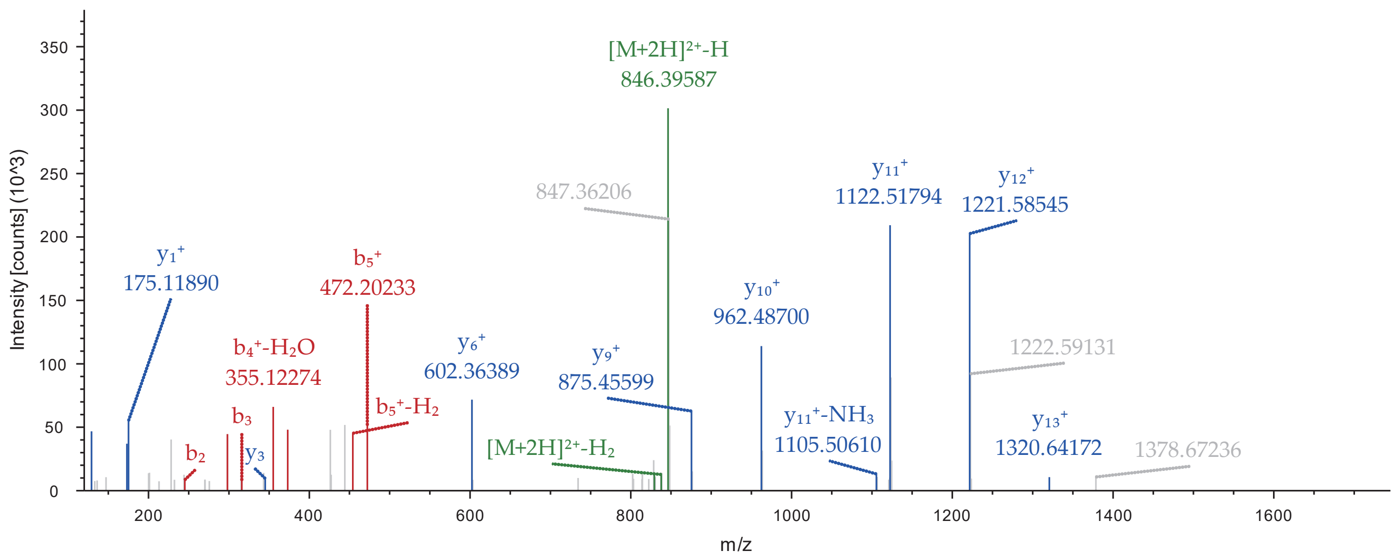

25. VCLDIQR

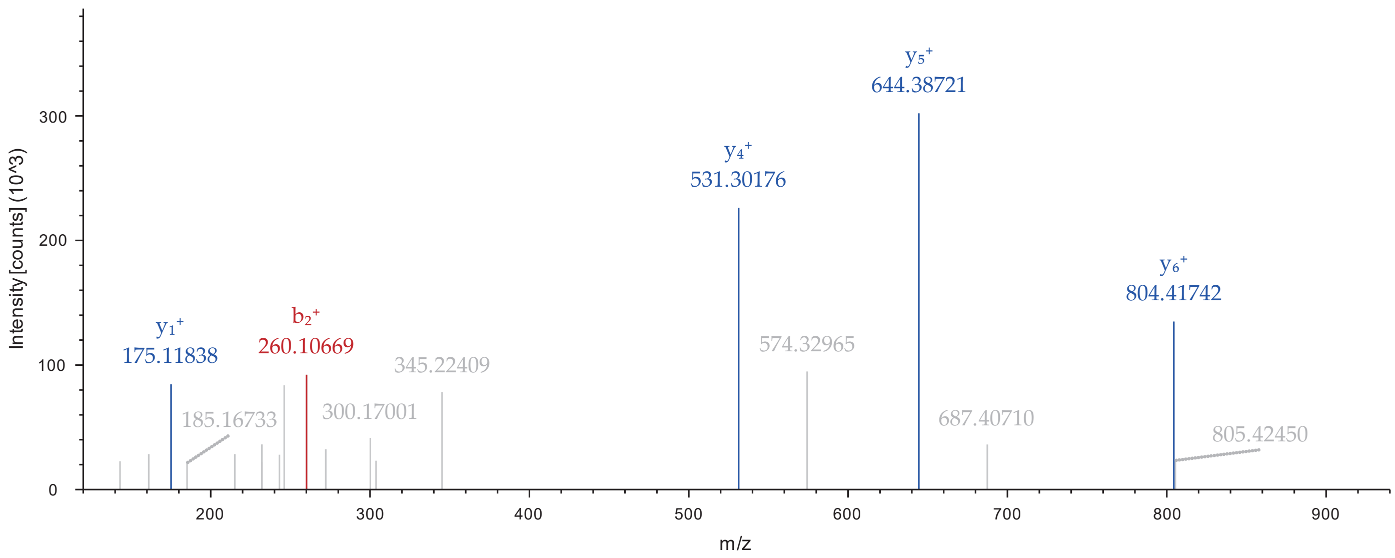

26. QPDSEAPDLNQLK

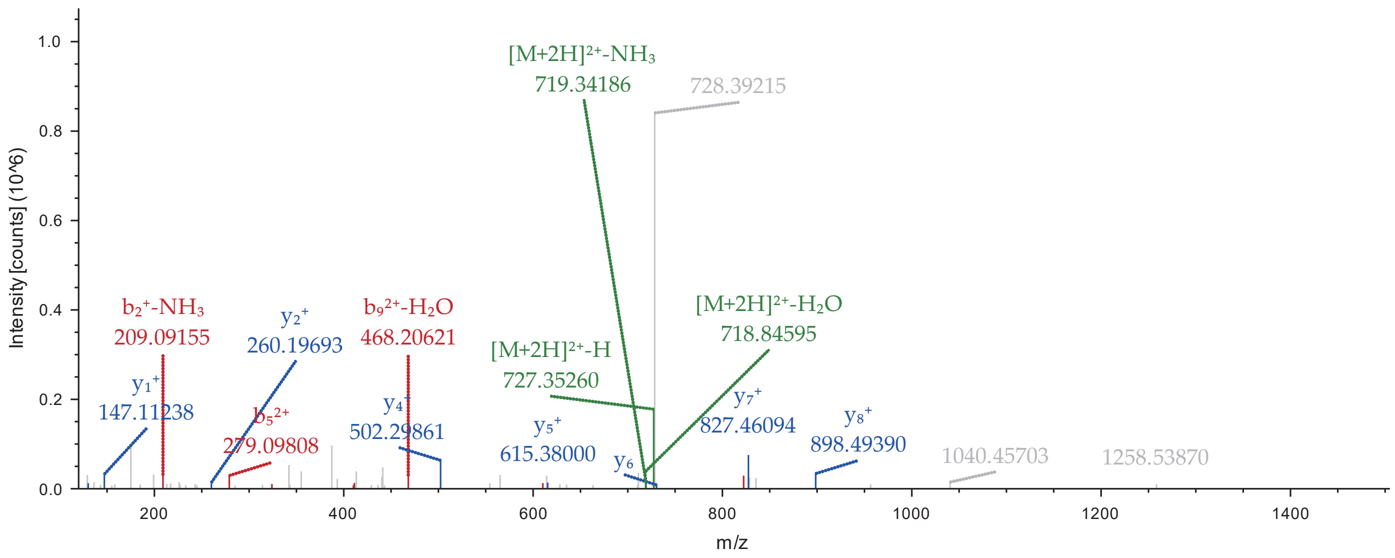

27. LAIAWR

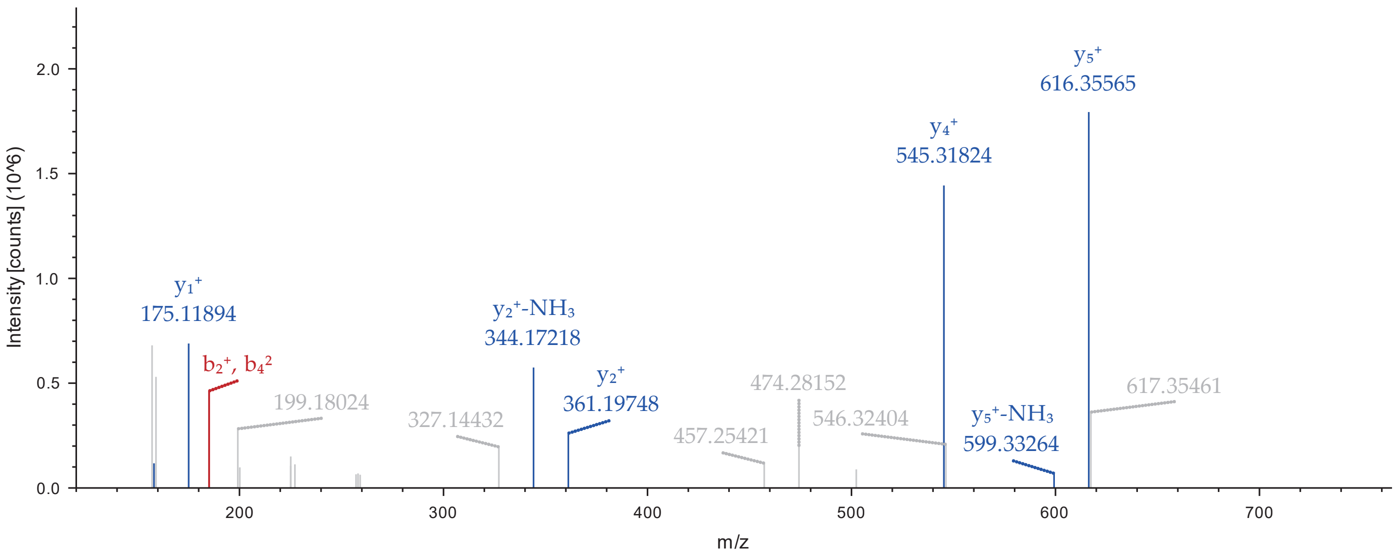

28. LTICLLR

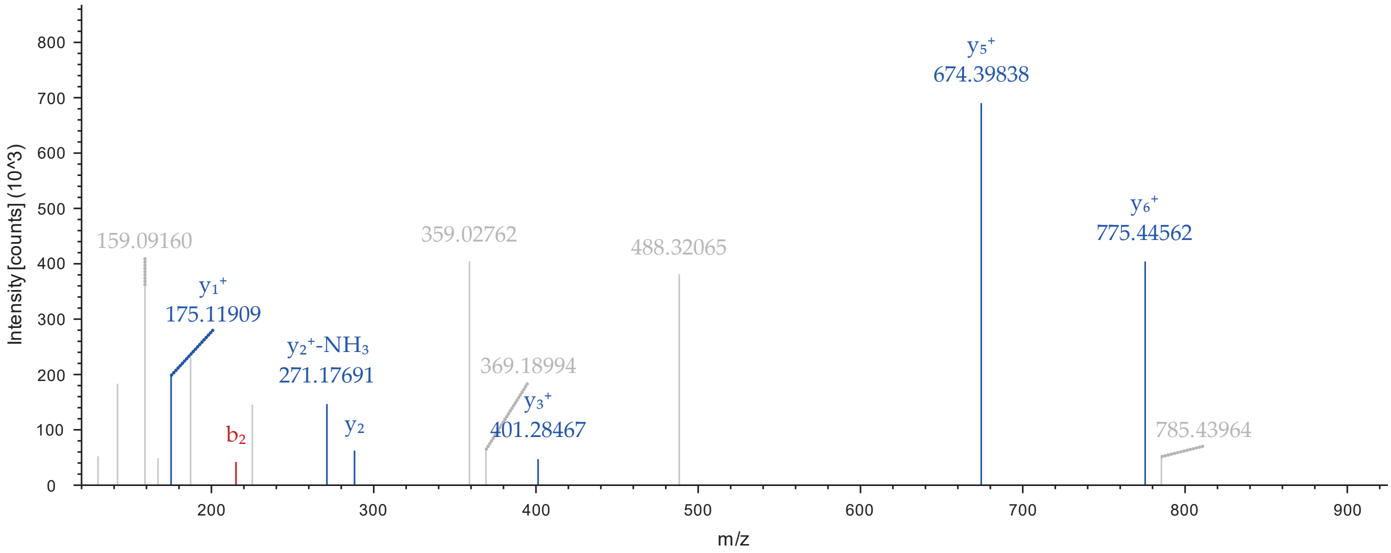

29. YSSNLSNFNYEQR

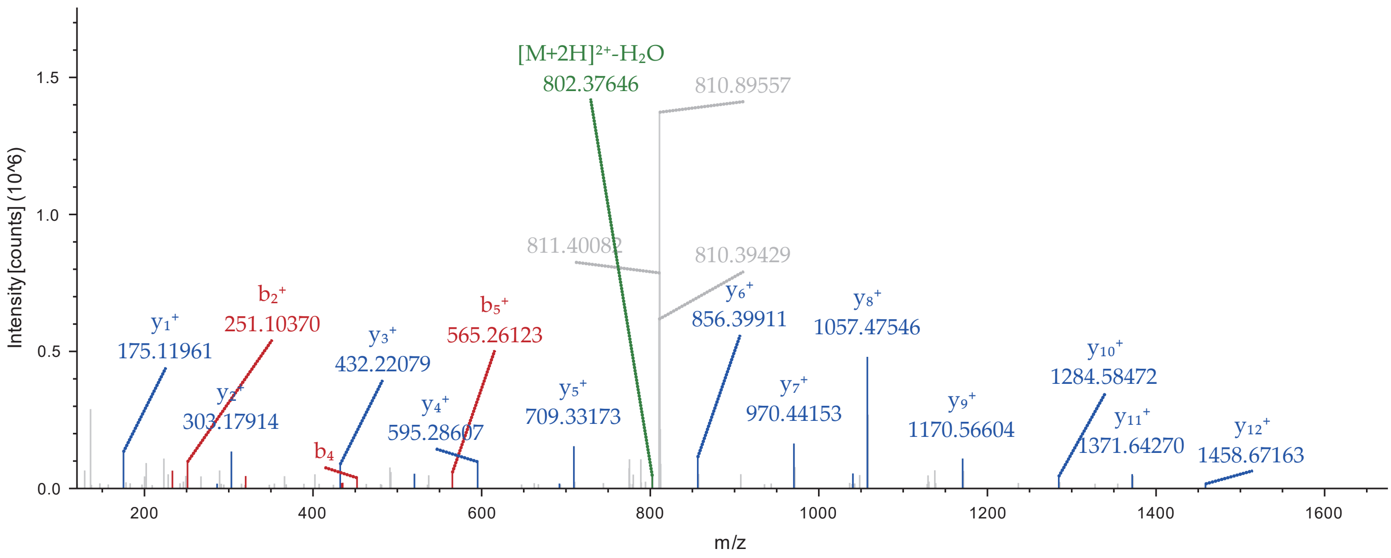

30. WTTEEQLLAVQGVR

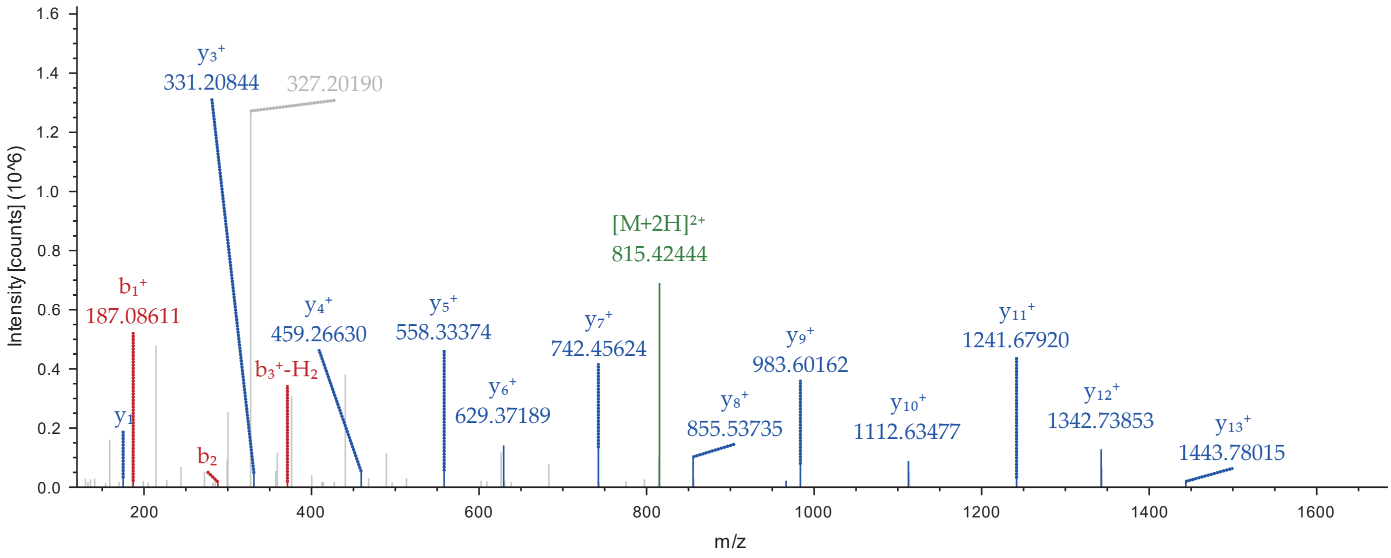

31. LGVSMAPVK

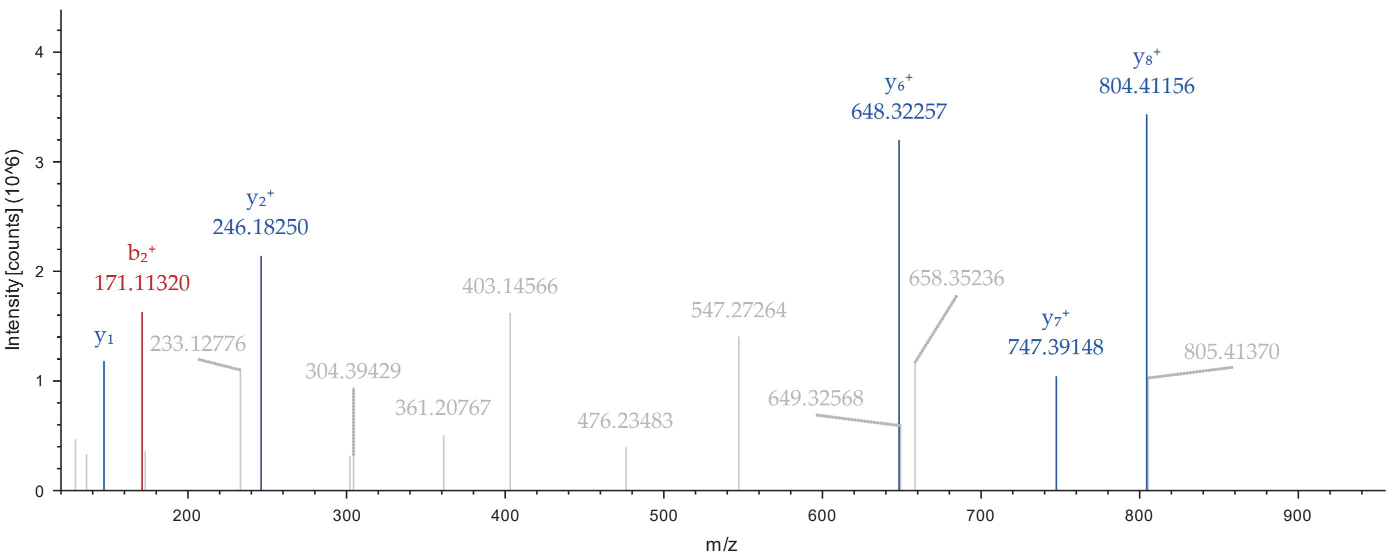

32. AHNAASEPAGGIAER

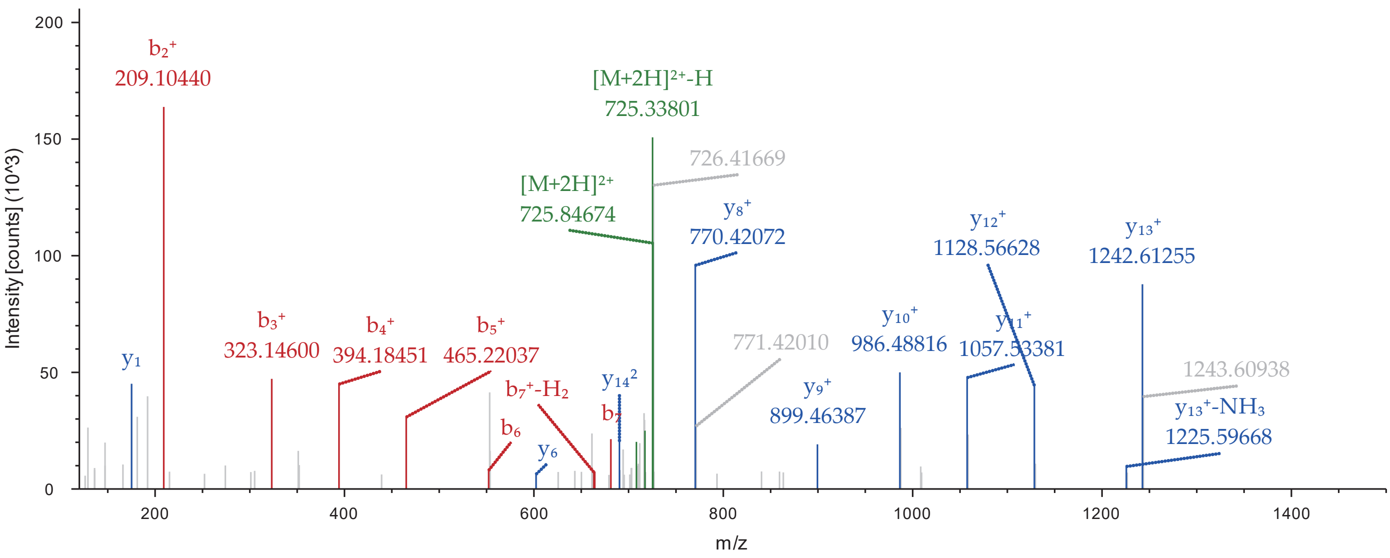

33. TPYDESQFYIGCDR

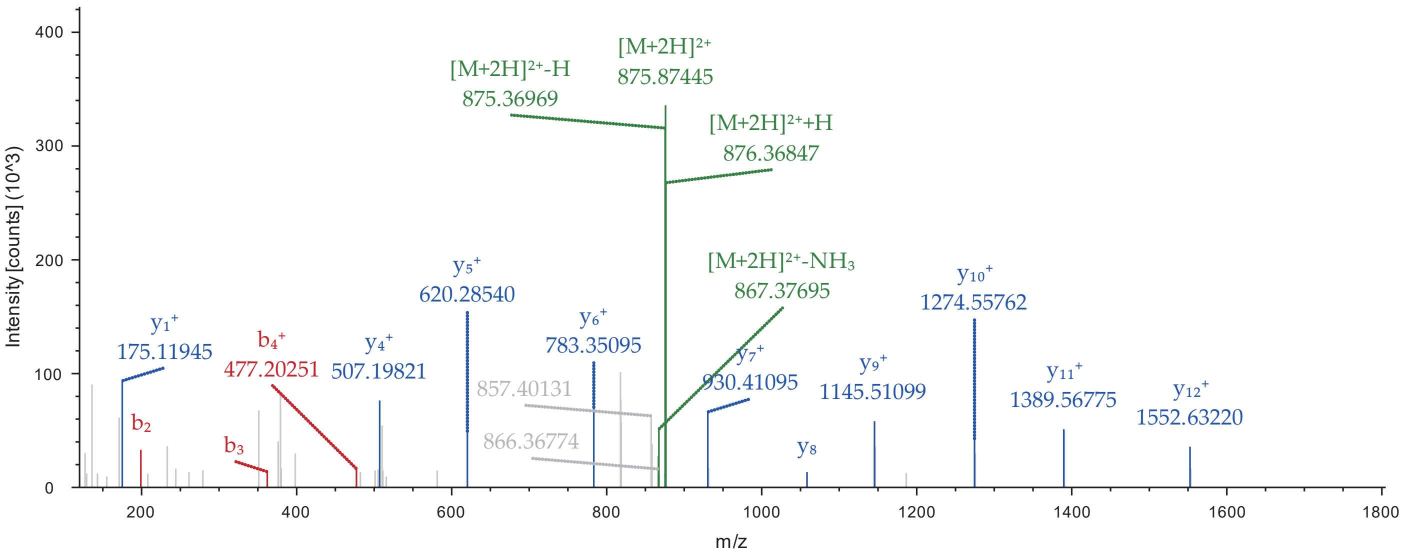

34. HLEILQQQLLQEQAMLLECR

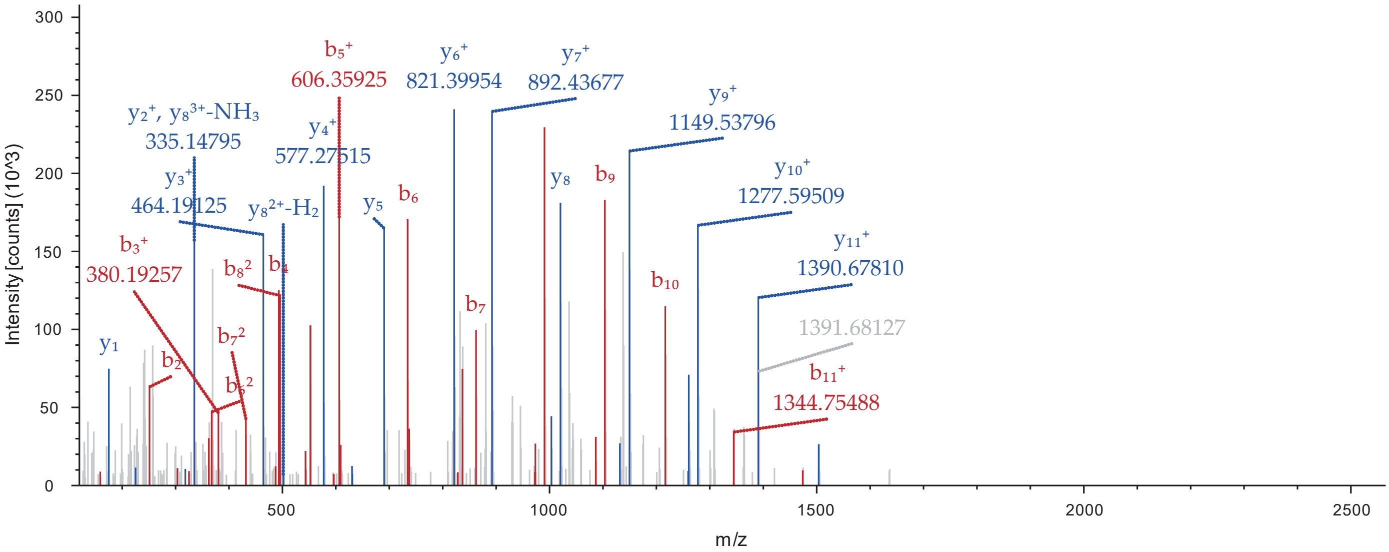

35. AAAGLMSLLR

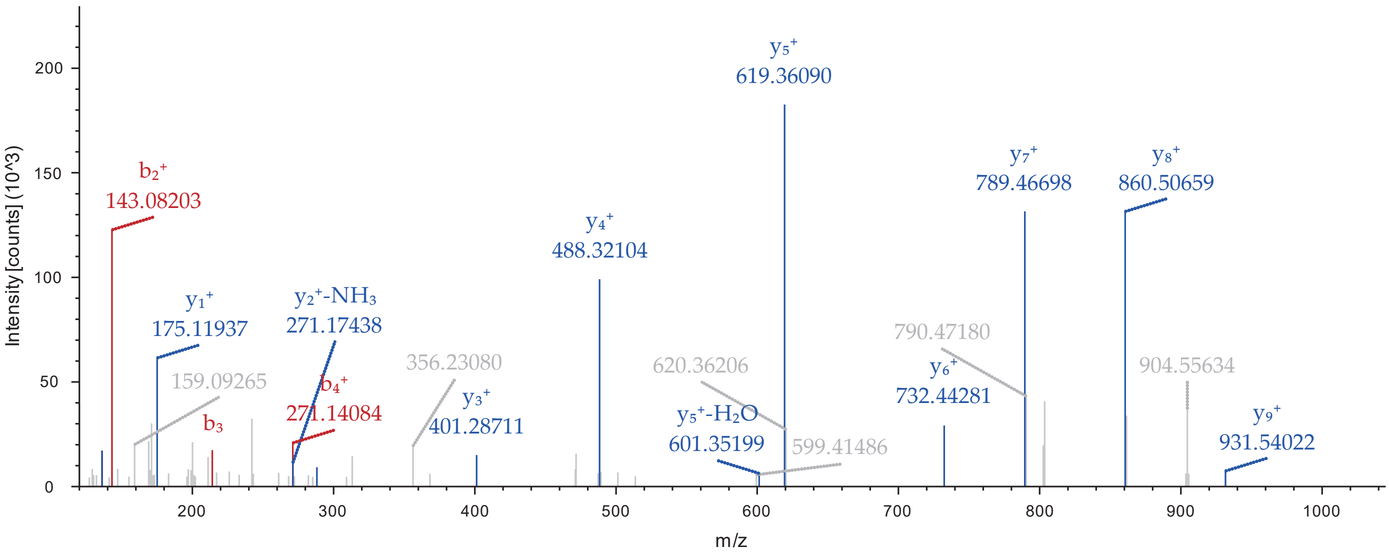

36. QGPSFAYGELEK

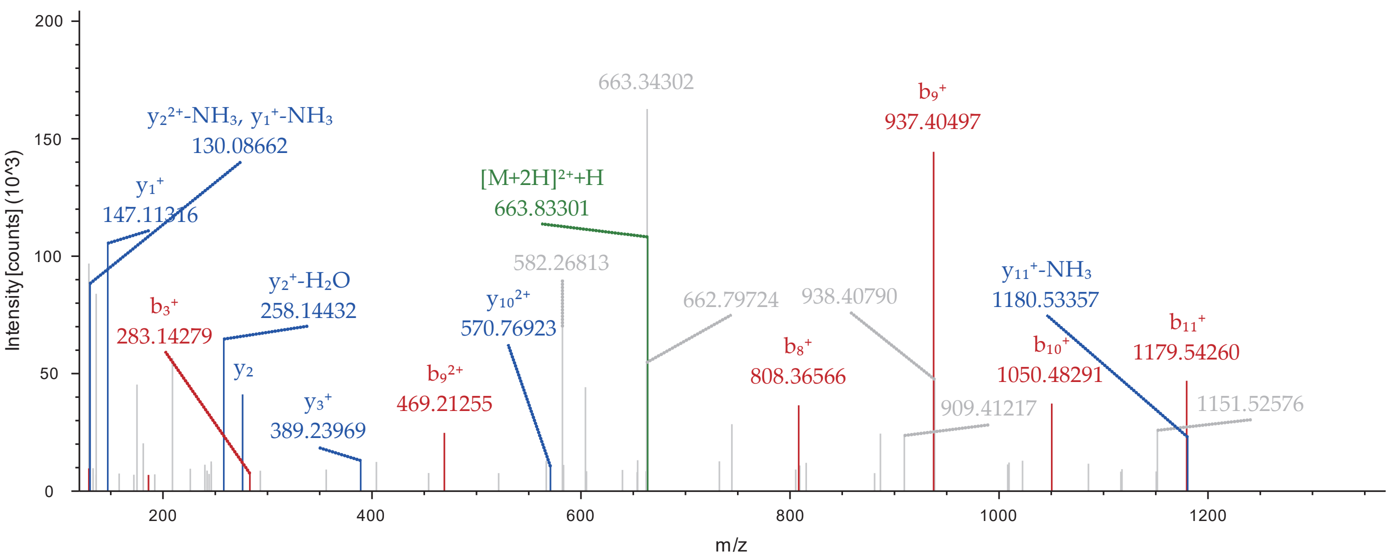

37. VPSALAPASQEPPPAASAEADGK

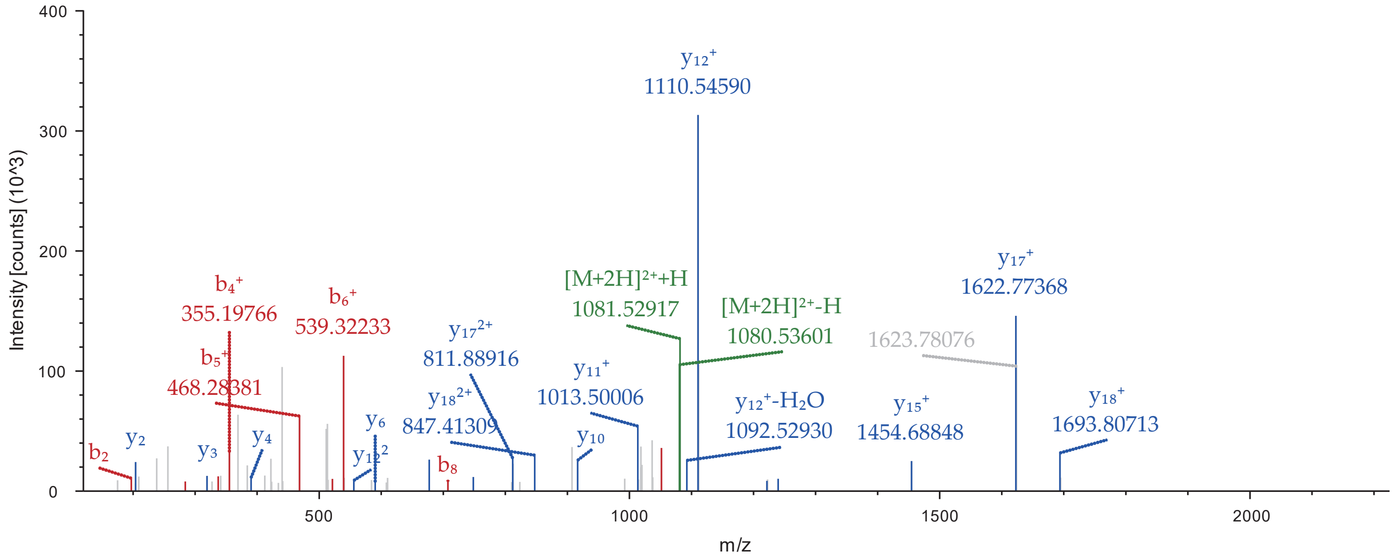

38. SLDGEVGTGGQYATTK

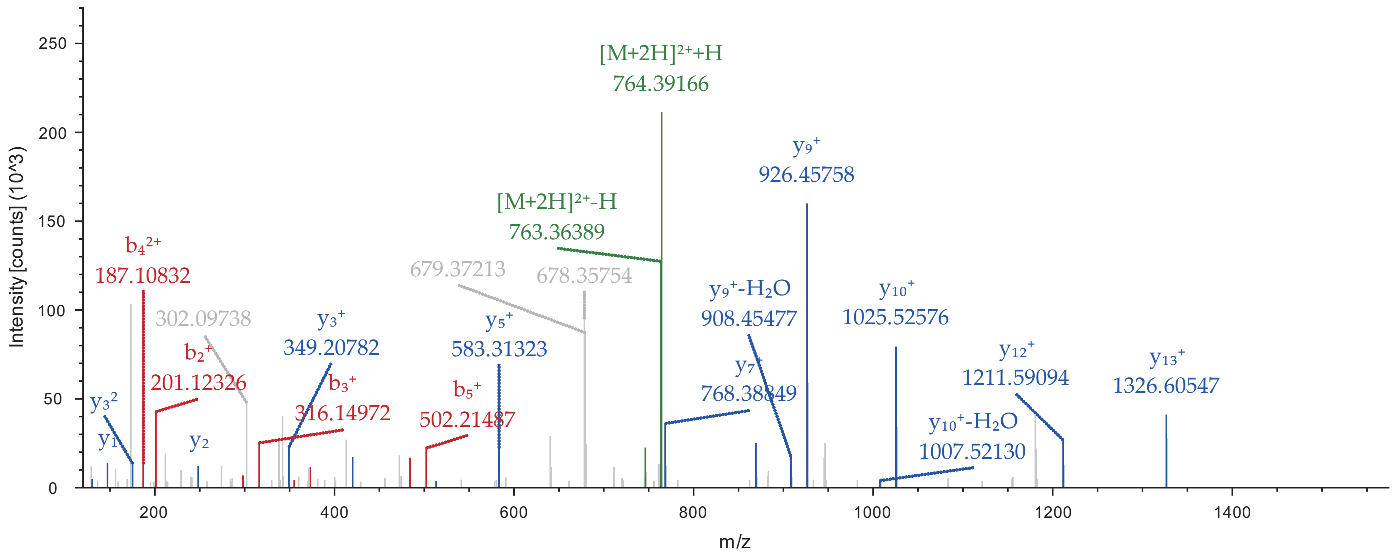

39. VNSGDTEVGSSLLR

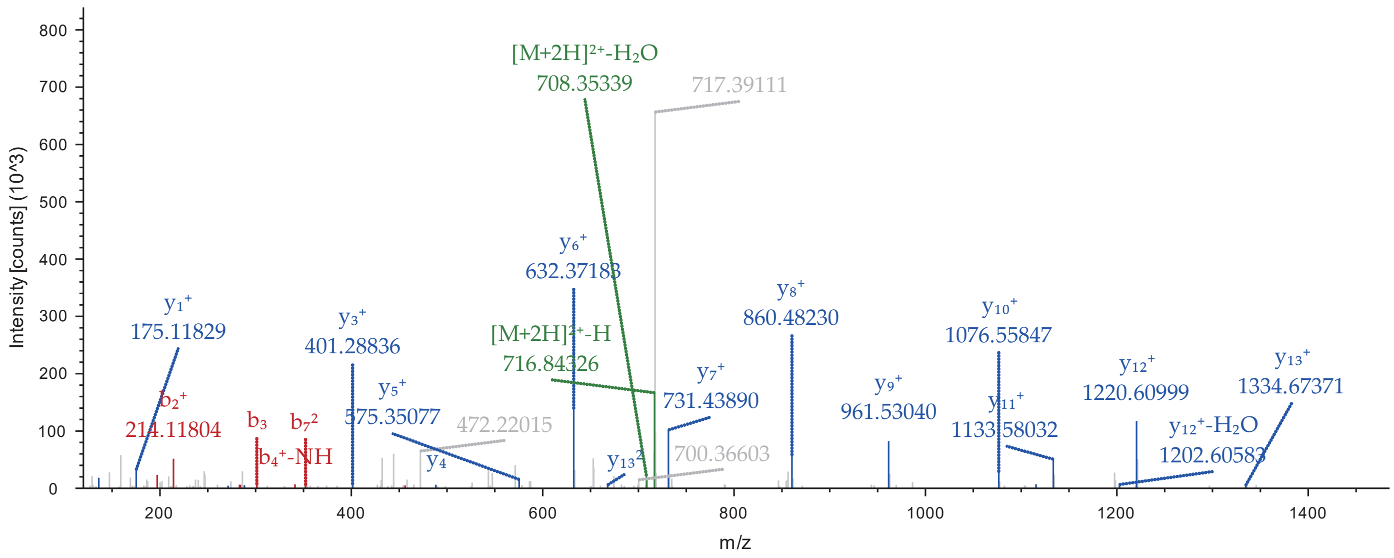

40. SCGADSQSENEASPVK

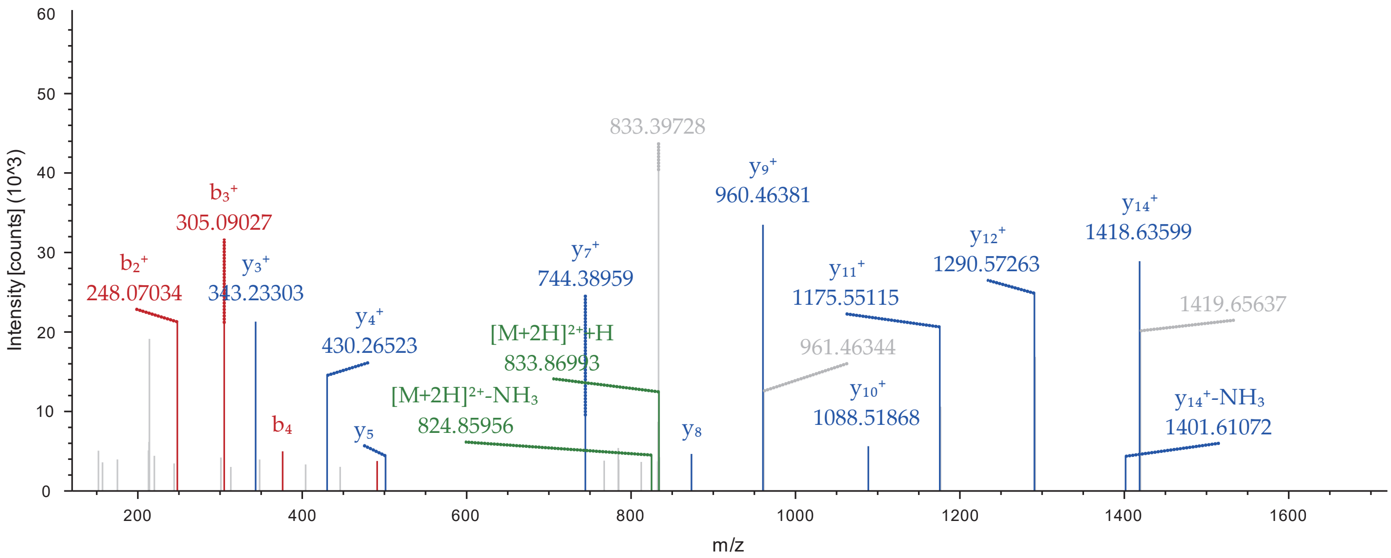

41. DQLGQELEELTASLFEEAHK

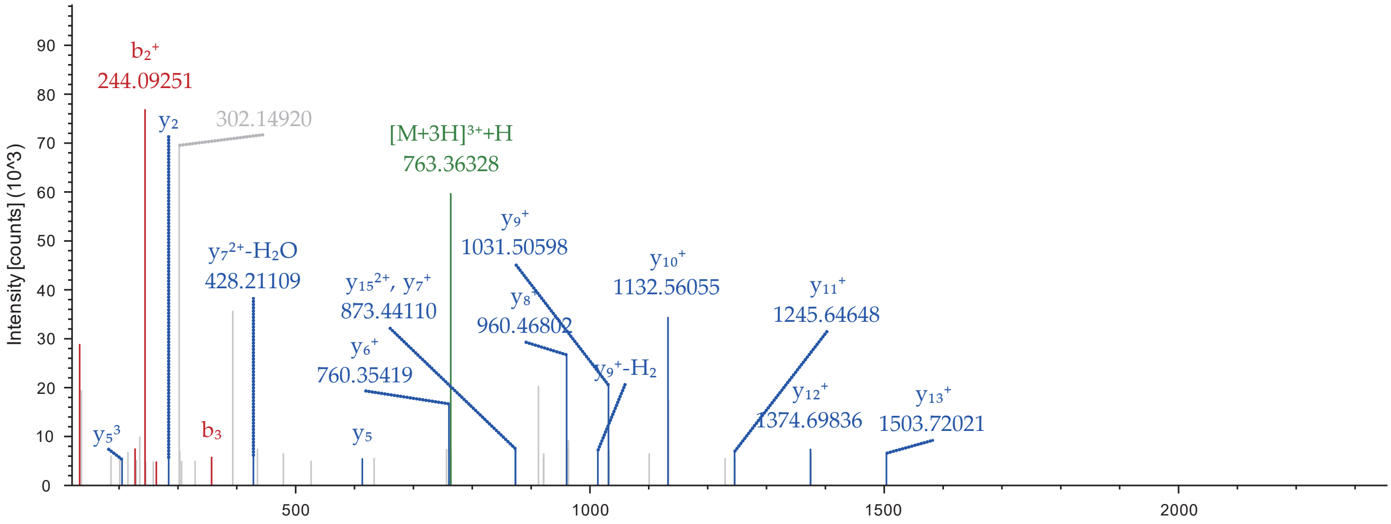

42. TAVVVPQSQAQPATAPQAAAAQEPGIQAPVR

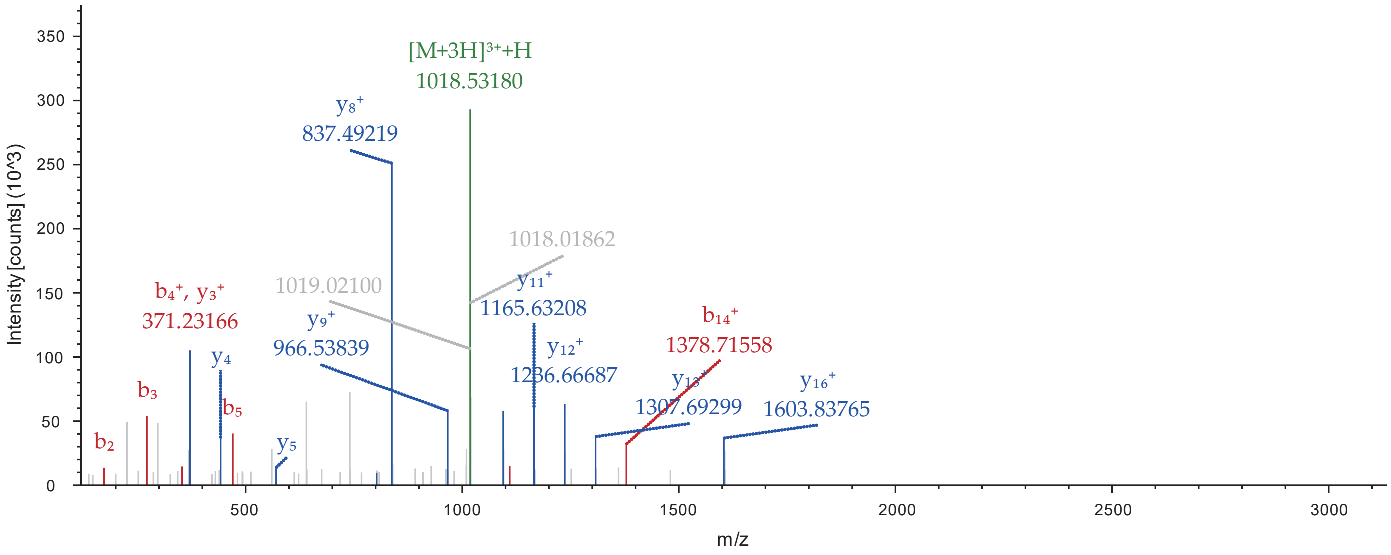

43. FLYGCQAPTICFVYQPLK

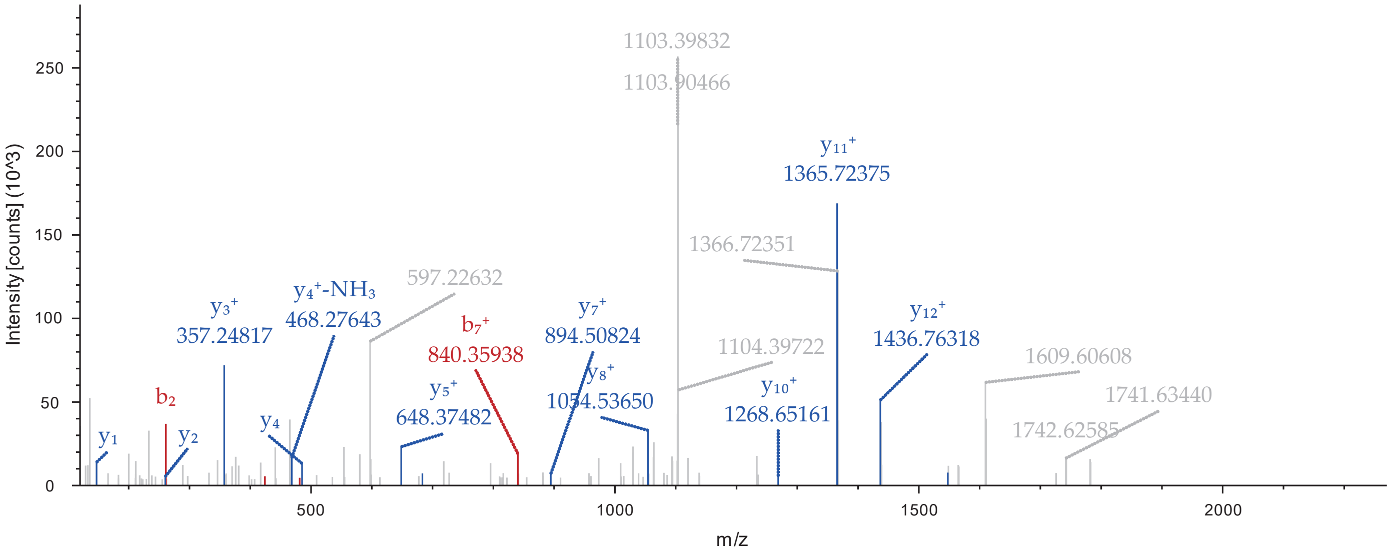

44. LQQEVSPALNSLQQTLK

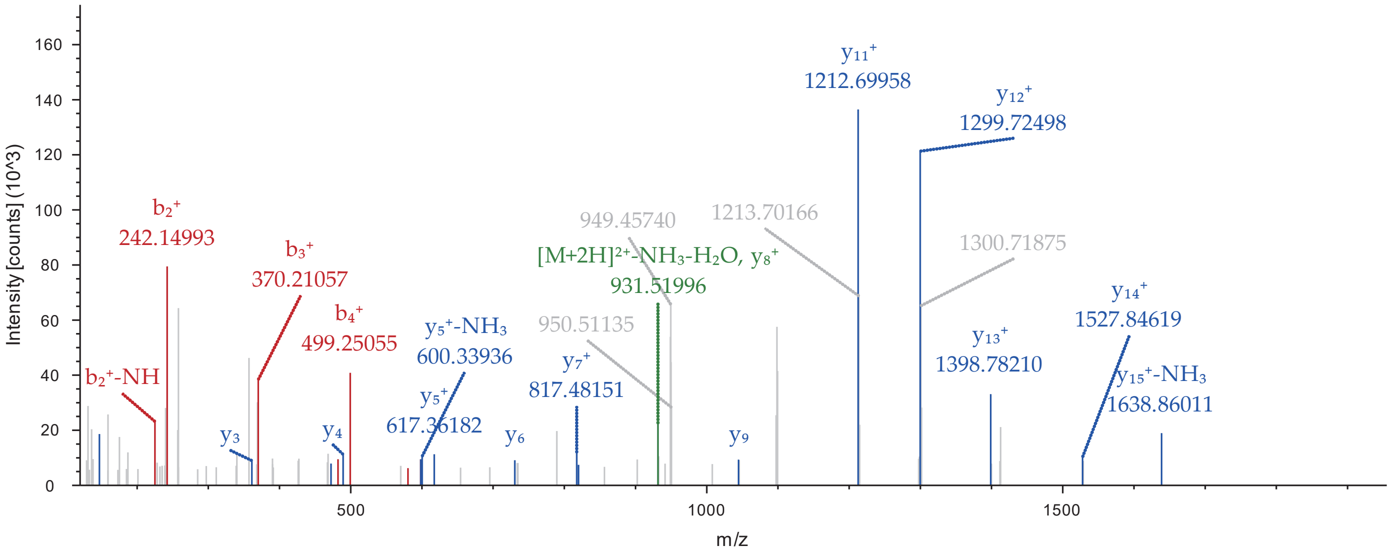

45. NTSGDPSSLESQMR

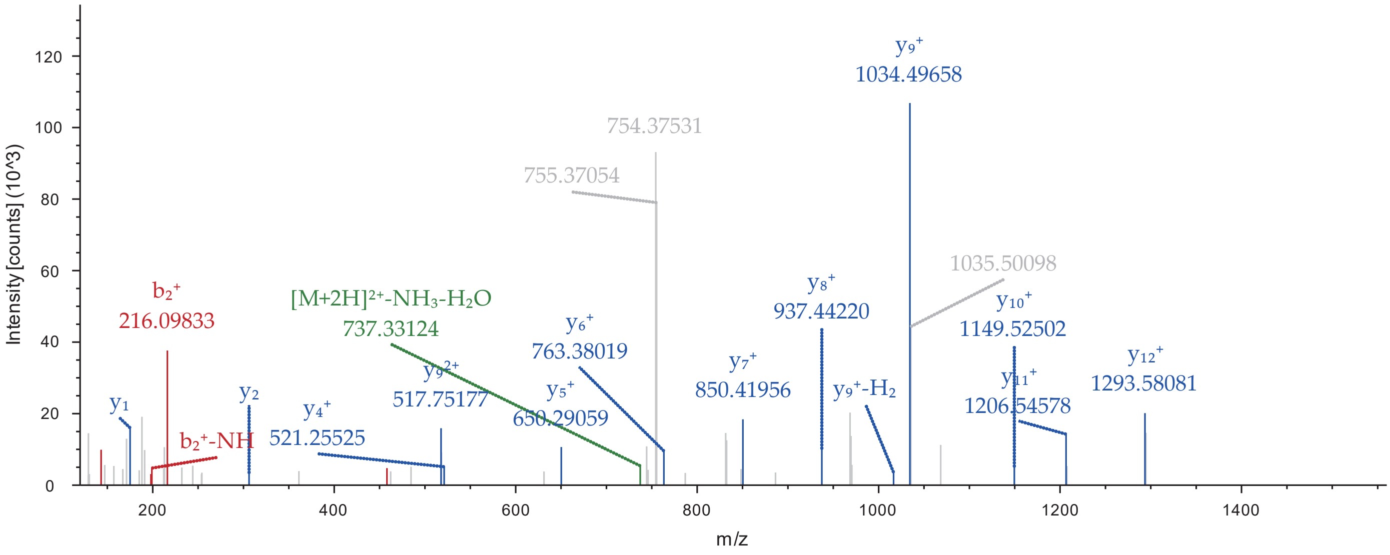

46. AAPAEVSSIIK

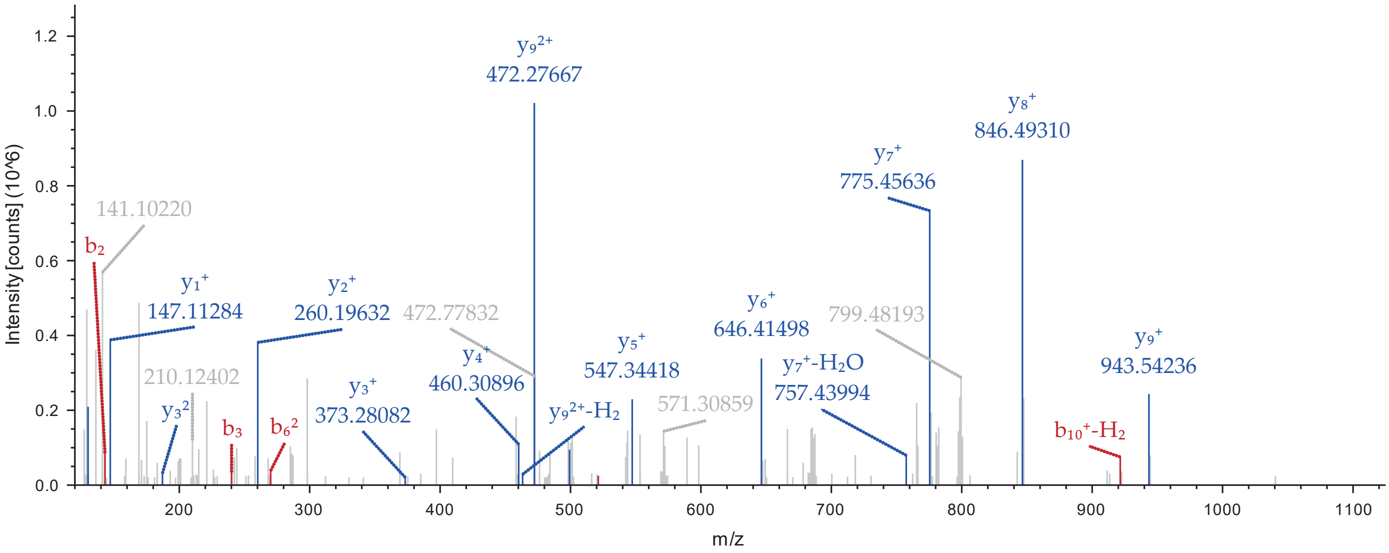

47. LIPVPANSYLGFSIDSGK

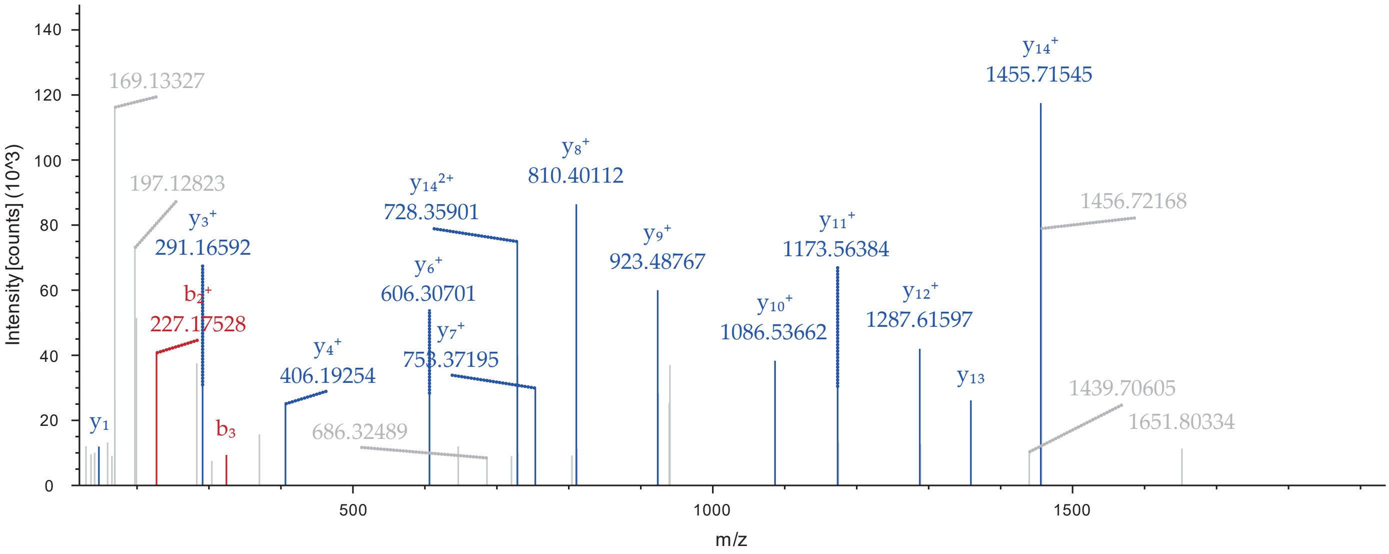

48. LLDGENIHSSSSQHSSGQSYSSR

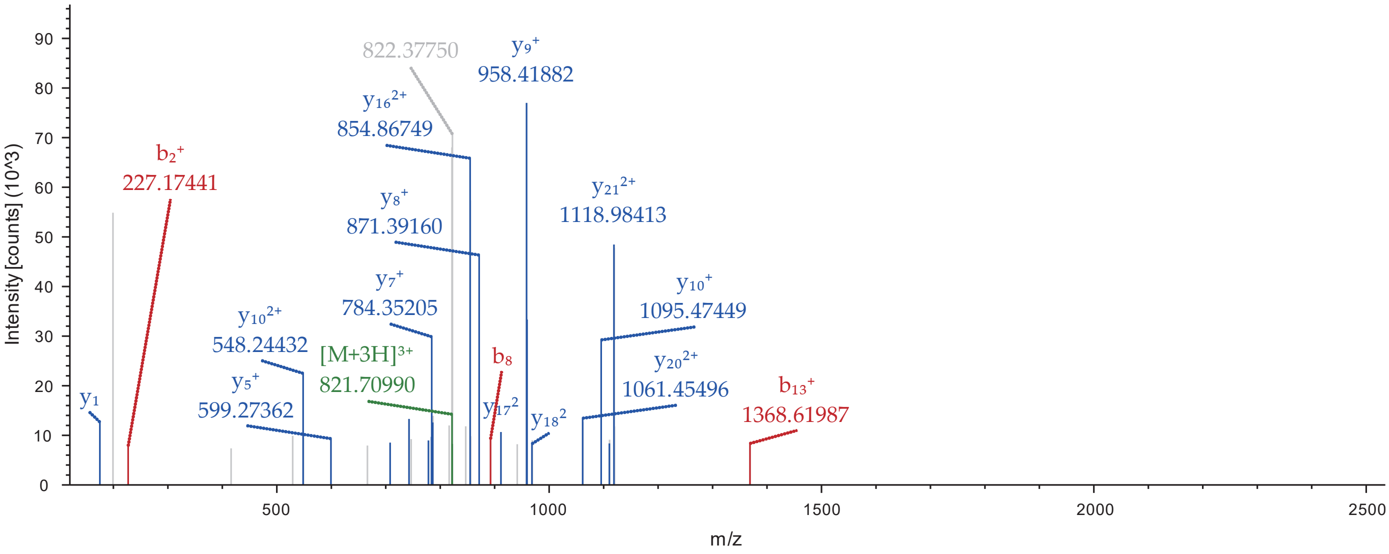

49. LQDEAYQLHVSTETR

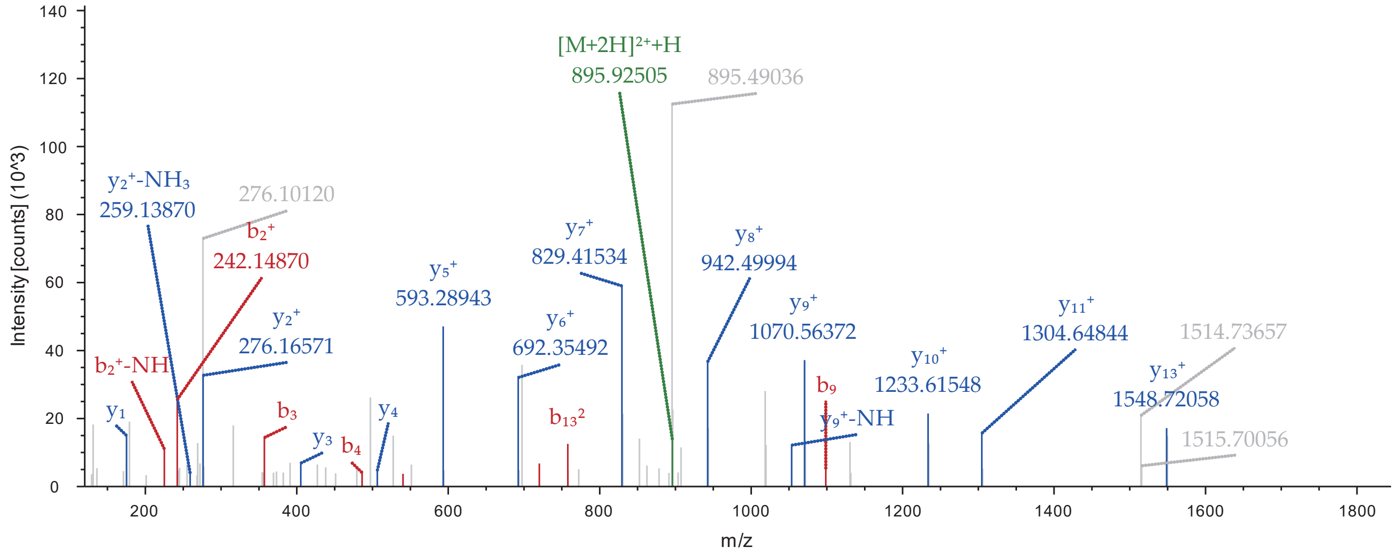

50. ILINFPR

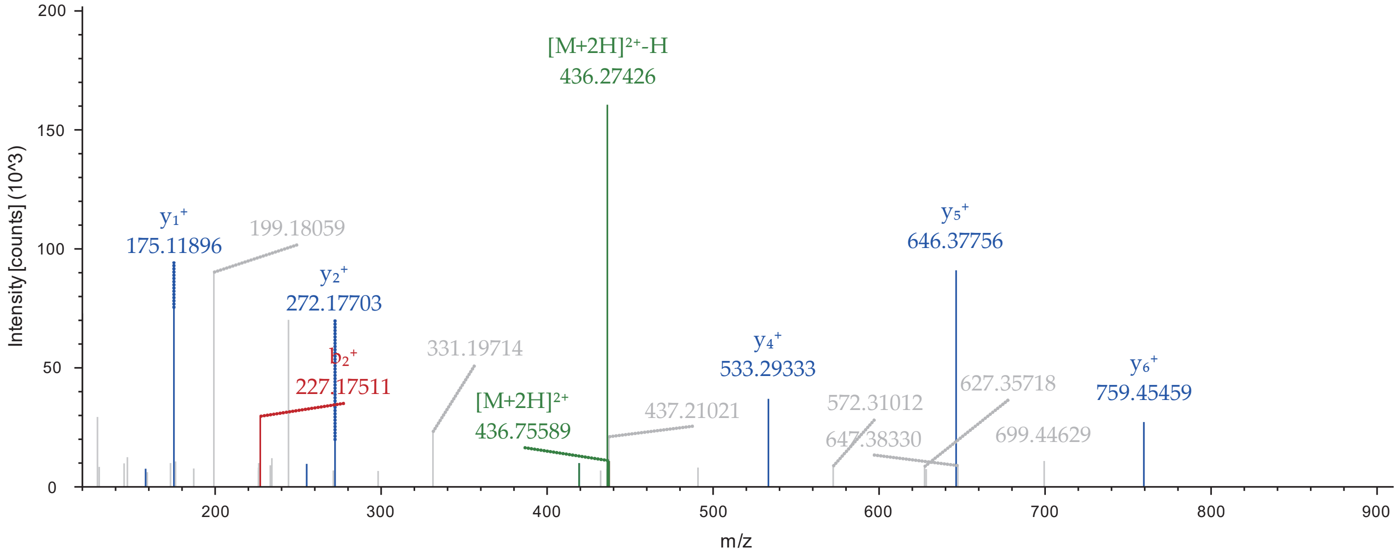

51. NNPGFPSLLR

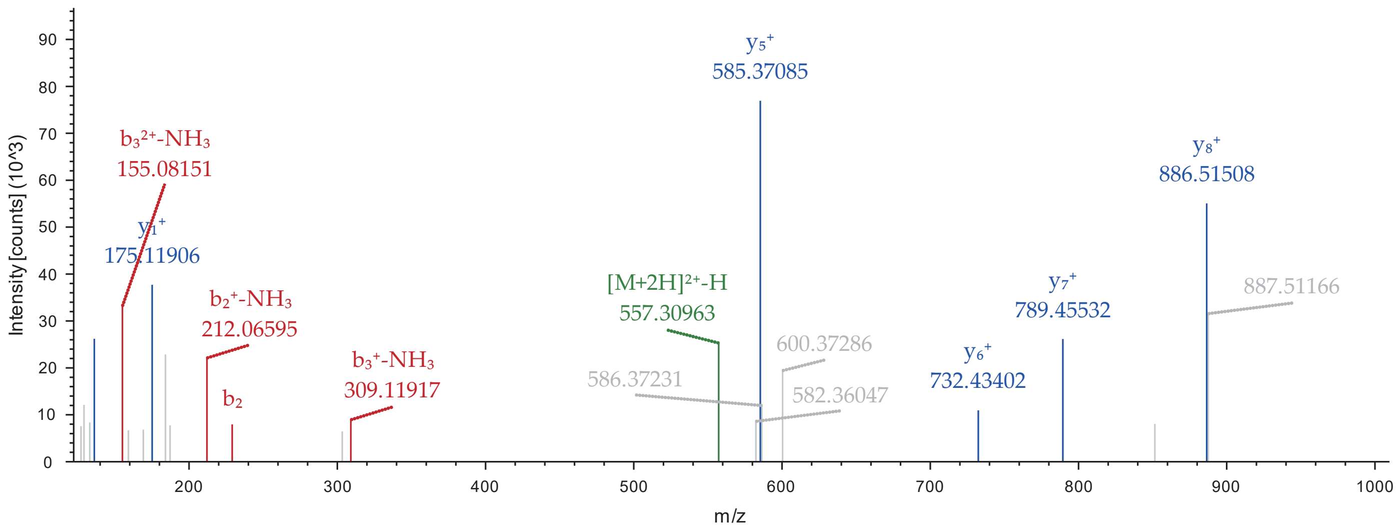

52. YPAPSLQSSVR

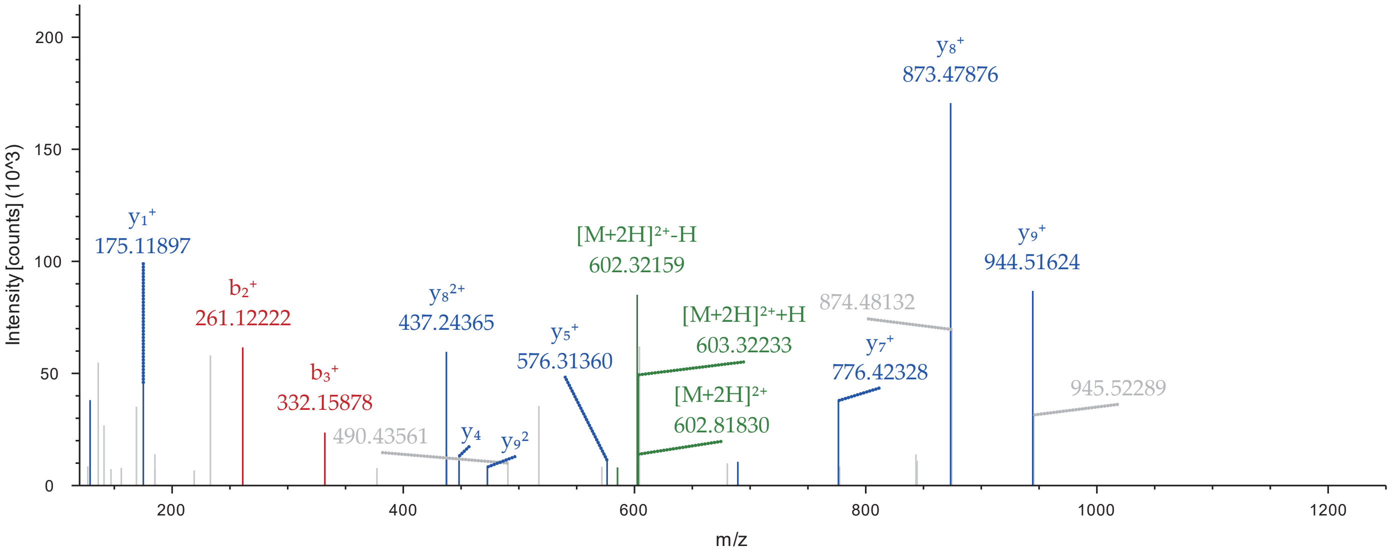

53. LGEGLQR

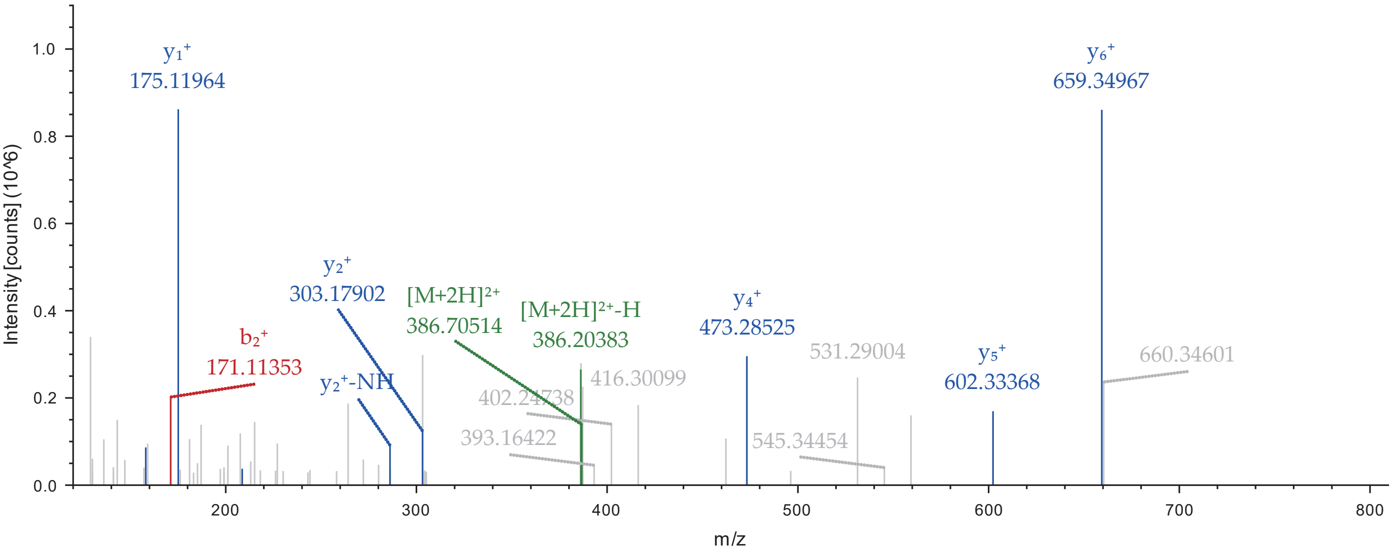

54. CLELEEMMEEQGYEEQQIQEK

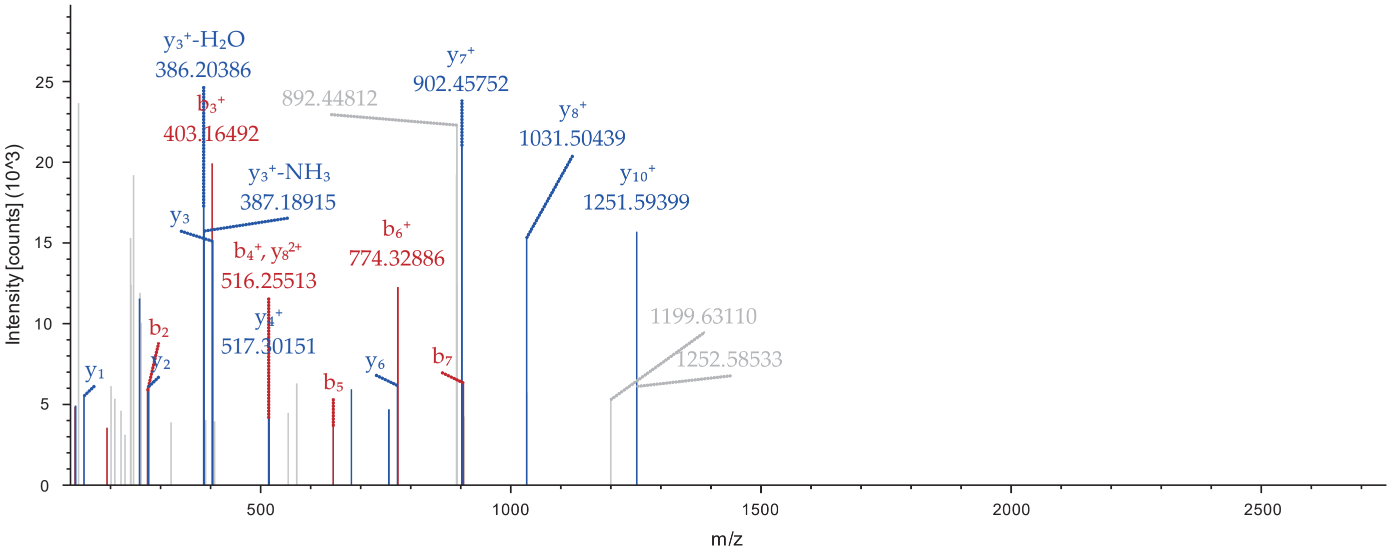

55. GLQGPLLLR

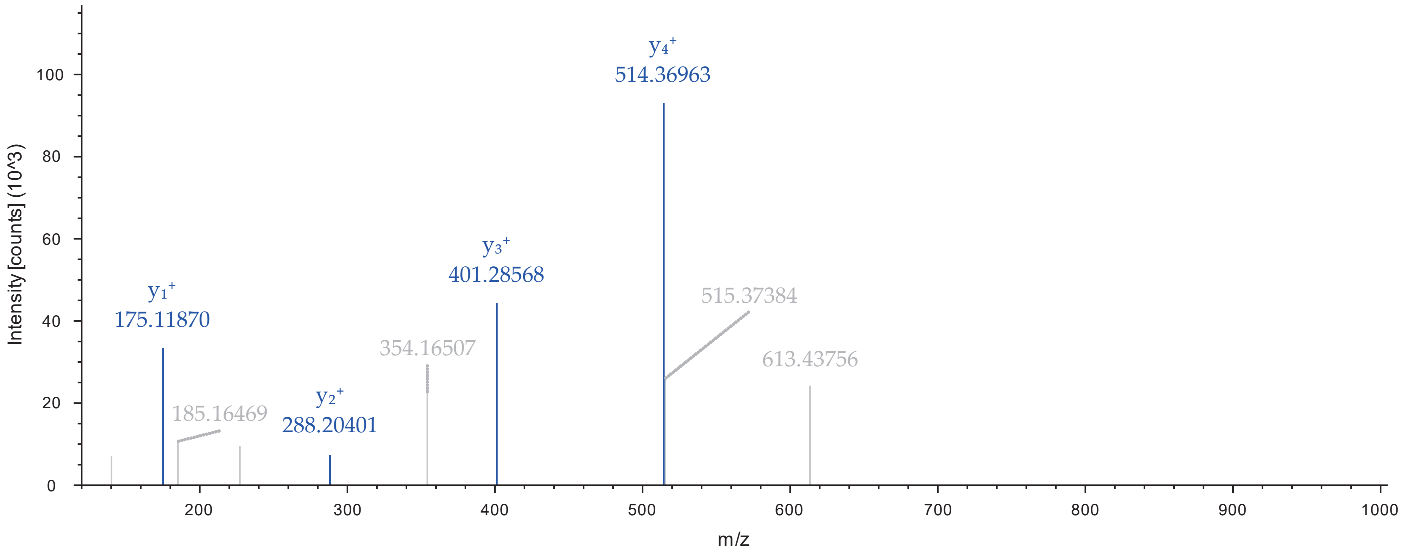

56. VGMGSGSICITQEAAPK

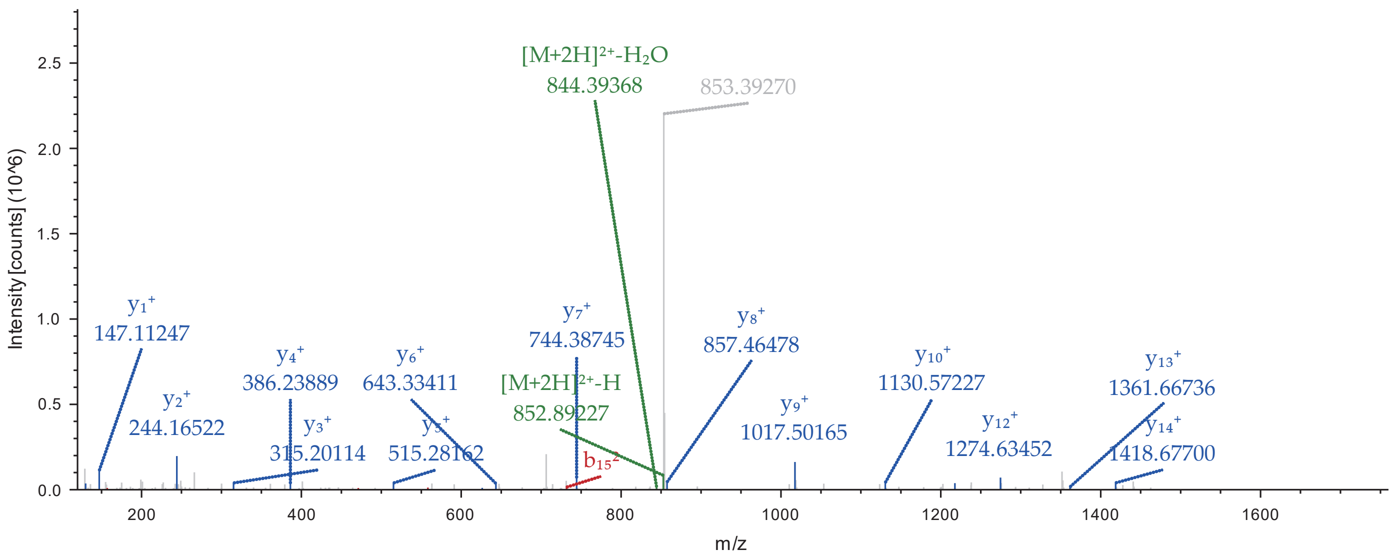

57. SLEQYDQDDPFK

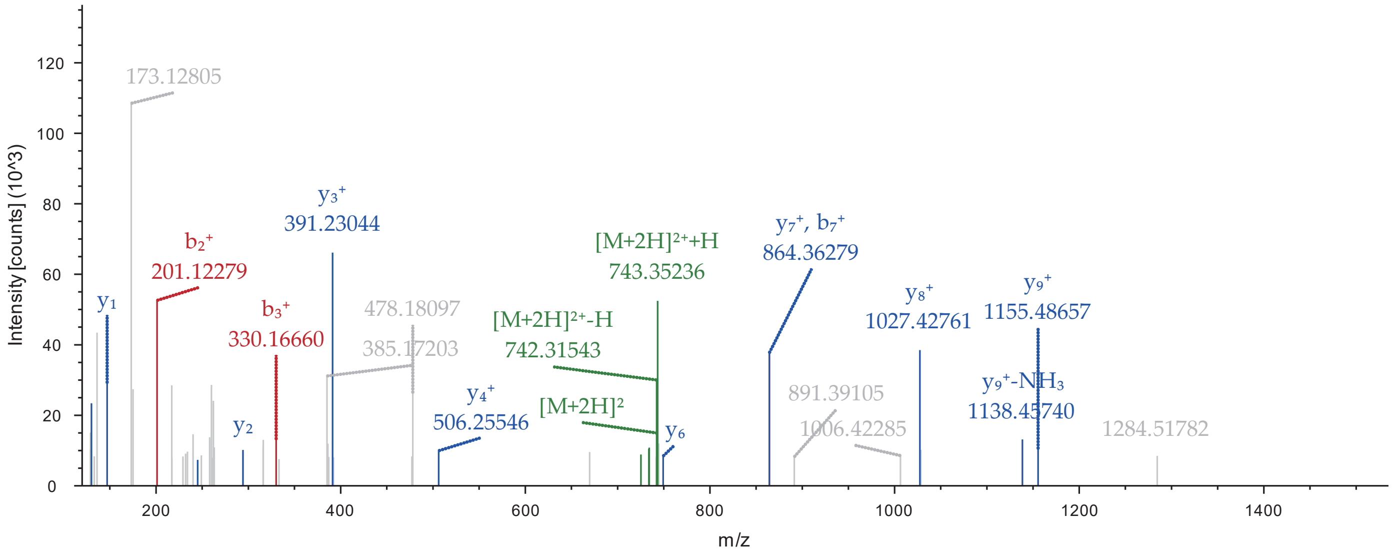

58. GAGAPSSPDSPTDVLMQLAK

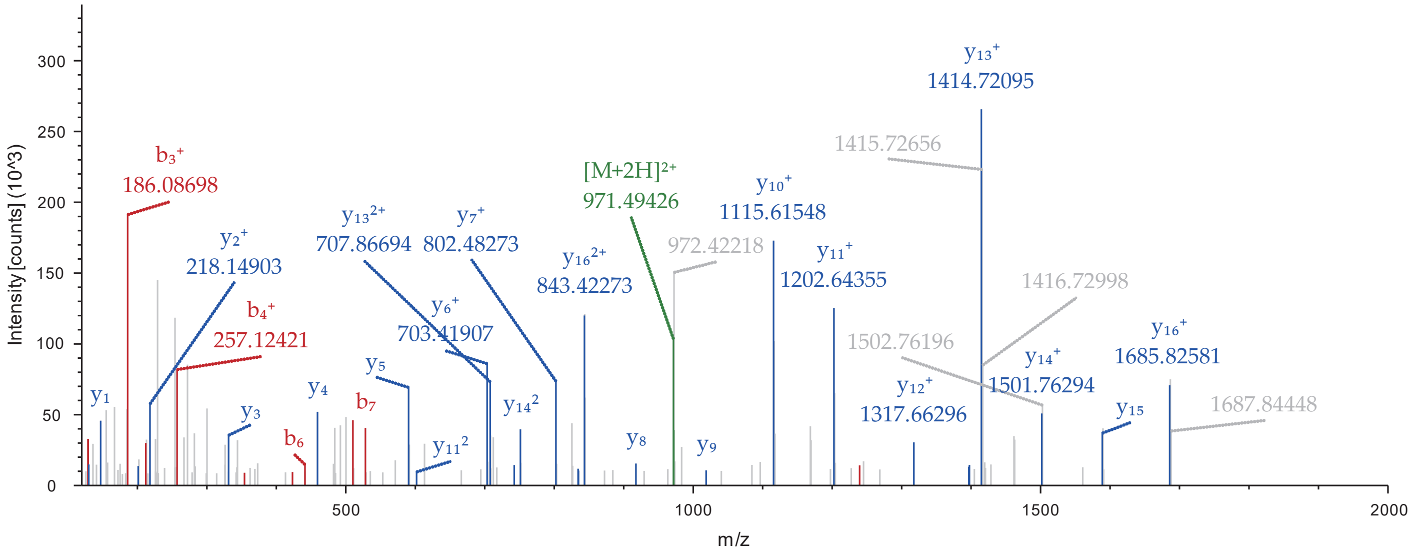

59. VAPALVTLSGDPEFSVR

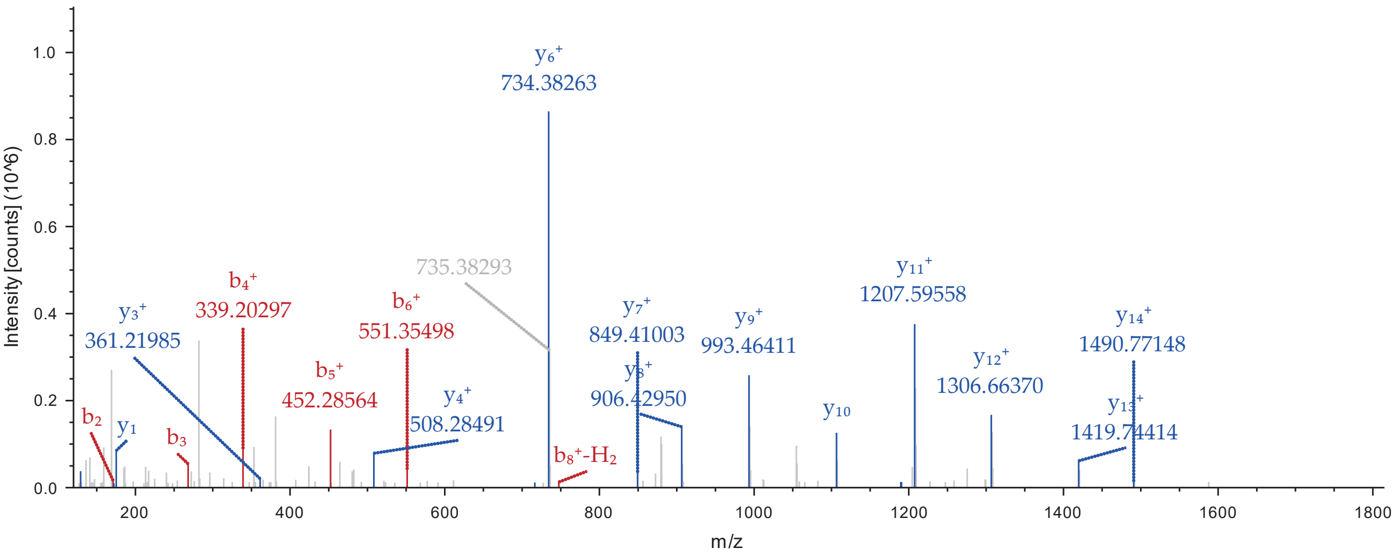

60. TTADEVTEK

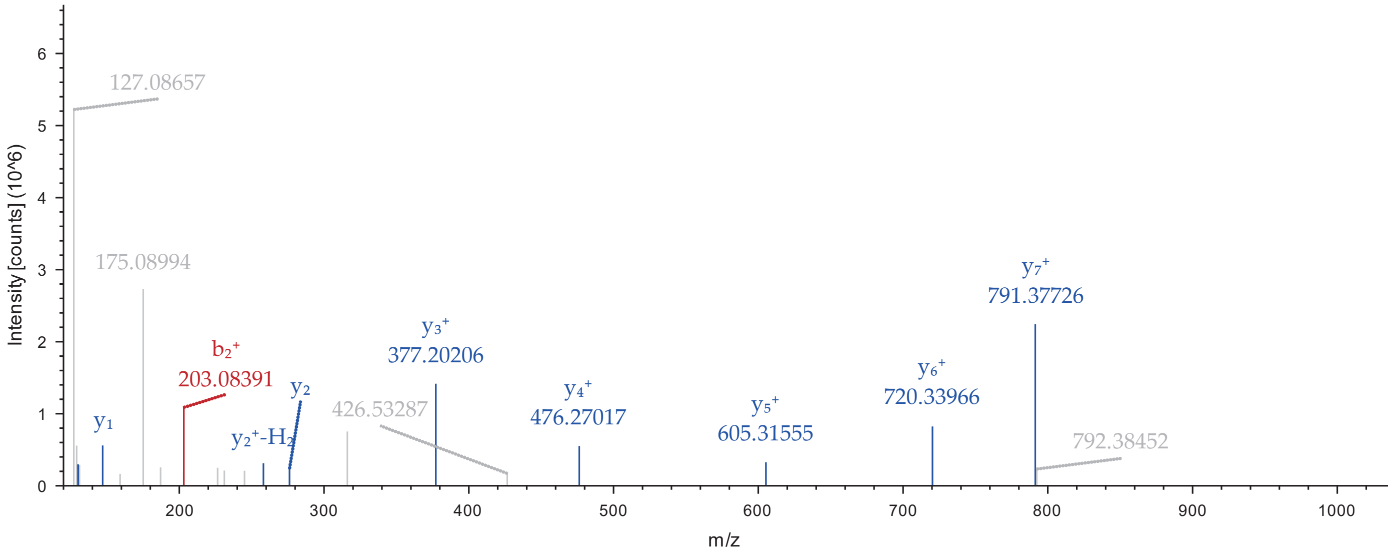

Supplement: Supplementary file 9 — Supplementary Data 6 [file 41467_2018_7463_MOESM9_ESM.pdf]
