# Supplementary material for: A time-resolved multi-omic atlas of the developing mouse stomach
Source: Nat Commun. 2018 Nov 21;9:4910. doi: 10.1038/s41467-018-07463-9 (PMC6249217; doi:10.1038/s41467-018-07463-9)
Supplement: Supplementary file 12 — Reporting Summary [file 41467_2018_7463_MOESM12_ESM.pdf]

nature research

Corresponding author(s): Yi Wang and Jun Qin

Reporting Summary

Nature Research wishes to improve the reproducibility of the work that we publish. This form provides structure for consistency and transparency in reporting. For further information on Nature Research policies, see Authors & Referees and the Editorial Policy Checklist.

Statistical parameters

When statistical analyses are reported, confirm that the following items are present in the relevant location (e.g. figure legend, table legend, main text, or Methods section).

n/a	Confirmed	
☐	☒	The exact sample size (n) for each experimental group/condition, given as a discrete number and unit of measurement
☐	☒	An indication of whether measurements were taken from distinct samples or whether the same sample was measured repeatedly
☐	☒	The statistical test(s) used AND whether they are one- or two-sided<br>*Only common tests should be described solely by name; describe more complex techniques in the Methods section.*
☒	☐	A description of all covariates tested
☒	☐	A description of any assumptions or corrections, such as tests of normality and adjustment for multiple comparisons
☐	☒	A full description of the statistics including central tendency (e.g. means) or other basic estimates (e.g. regression coefficient) AND variation (e.g. standard deviation) or associated estimates of uncertainty (e.g. confidence intervals)
☒	☐	For null hypothesis testing, the test statistic (e.g. F, t, r) with confidence intervals, effect sizes, degrees of freedom and P value noted<br>*Give P values as exact values whenever suitable.*
☒	☐	For Bayesian analysis, information on the choice of priors and Markov chain Monte Carlo settings
☐	☒	For hierarchical and complex designs, identification of the appropriate level for tests and full reporting of outcomes
☐	☒	Estimates of effect sizes (e.g. Cohen's d, Pearson's r), indicating how they were calculated
☐	☒	Clearly defined error bars<br>*State explicitly what error bars represent (e.g. SD, SE, CI)*

Our web collection on statistics for biologists may be useful.

Software and code

Policy information about availability of computer code

Data collection	R version 3.4.4, python 3.4, DAVID 6.8, FastQC 0.11.5, HISAT2 2.1.0, StringTie 1.3.1
Data analysis	Principal component analysis (PCA) and unsupervised hierarchical clustering analysis were performed on 7,678 GPs in Dataset3 and grouped 15 timepoints into three phases. Gene Ontology (GO) term enrichment analysis was based on DAVID (the Database for Annotation, Visualization and Integrated Discovery) Bioinformatics Resources. Differentially expressed genes in the three phases were identified by One-way ANOVA test with Benjamini-Hochberg adjustment. Gene coexpression analysis was conducted by Ward's hierarchical clustering analysis. Interexperiment correlations were calculated by Spearman's correlation coefficients. Two-tailed hypergeometric statistical tests were carried out to determine whether there is a significant overlap of altered genes on a specific pathway between stomach development and gastric cancer.

For manuscripts utilizing custom algorithms or software that are central to the research but not yet described in published literature, software must be made available to editors/reviewers upon request. We strongly encourage code deposition in a community repository (e.g. GitHub). See the Nature Research guidelines for submitting code & software for further information.

Data

Policy information about availability of data

All manuscripts must include a data availability statement. This statement should provide the following information, where applicable:

- Accession codes, unique identifiers, or web links for publicly available datasets
- A list of figures that have associated raw data
- A description of any restrictions on data availability

MS raw files and searching output data are deposited into proteomeXchange with the accession number PXD010702 (http://www.iprox.org/page/PDV014.html?projectId=IPX0001258000); RNA-Seq data are deposited into the NCBI Gene Expression Omnibus (GEO) database (GEO accession # GSE118083, https://www.ncbi.nlm.nih.gov/geo/query/acc.cgi?acc=GSE118083).

Field-specific reporting

Please select the best fit for your research. If you are not sure, read the appropriate sections before making your selection.

☒ Life sciences ☐ Behavioural & social sciences ☐ Ecological, evolutionary & environmental sciences

For a reference copy of the document with all sections, see nature.com/authors/policies/ReportingSummary-flat.pdf

Life sciences study design

All studies must disclose on these points even when the disclosure is negative.

Sample size	At E12.5-E14.5, we collected three embryo mice stomachs and used in one replicate; at E15.5-E17.5, two embryo mice stomachs were collected and combined into one replicate; after E18.5, only one mouse stomach was used in one replicate. At least three replicates were carried out at each time point.
Data exclusions	First, we excluded the experiments that had less than 5,000 protein IDs and only retained the 3 replicates with the best correlation. We then chose confident protein IDs with the following criteria: i) at 1% peptide FDR; ii) identified with at least one unique peptide and two strict peptides; iii) identified at least twice out of three replicates in at least one time point.
Replication	All attempts at replication were successful.
Randomization	Male and female mice mated randomly and the stomachs were allocated into different time points according to their offspring's development stages.
Blinding	The investigators were not blinded to group allocation during data collection and analysis. The purpose of the manuscript is to investigate the gene expression profiles of mouse stomach from embryo to adult according to the actual developmental time point.

Reporting for specific materials, systems and methods

Materials & experimental systems

n/a	Involved in the study
☒	☐ Unique biological materials
☒	☐ Antibodies
☒	☐ Eukaryotic cell lines
☒	☐ Palaeontology
☐	☒ Animals and other organisms
☒	☐ Human research participants

Methods

n/a	Involved in the study
☒	☐ ChIP-seq
☒	☐ Flow cytometry
☒	☐ MRI-based neuroimaging

Animals and other organisms

Policy information about studies involving animals; ARRIVE guidelines recommended for reporting animal research

Laboratory animals	Male and female C57BL/6 mice (8-10 weeks old) were mated randomly and their offspring were used to obtain stomach tissues from E12.5 to eight weeks old. Stomachs obtained from the offspring within 2 weeks were without gender distinction while others were obtained from male mice.

Wild animals	The study did not involve wild animals.
Field-collected samples	The study did not involve samples collected from the field.
